# New Trends in Aging Drug Discovery

**DOI:** 10.3390/biomedicines10082006

**Published:** 2022-08-18

**Authors:** Bellinda Benhamú, Mar Martín-Fontecha, Henar Vázquez-Villa, María L. López-Rodríguez, Silvia Ortega-Gutiérrez

**Affiliations:** 1 Departamento de Química Orgánica, Facultad de Ciencias Químicas, Universidad Complutense de Madrid, 28040 Madrid, Spain; 2 Departamento de Química Orgánica, Facultad de Óptica y Optometría, Universidad Complutense de Madrid, 28037 Madrid, Spain

**Keywords:** aging, drug discovery, senescence

## Abstract

Aging is considered the main risk factor for many chronic diseases that frequently appear at advanced ages. However, the inevitability of this process is being questioned by recent research that suggests that senescent cells have specific features that differentiate them from younger cells and that removal of these cells ameliorates senescent phenotype and associated diseases. This opens the door to the design of tailored therapeutic interventions aimed at reducing and delaying the impact of senescence in life, that is, extending healthspan and treating aging as another chronic disease. Although these ideas are still far from reaching the bedside, it is conceivable that they will revolutionize the way we understand aging in the next decades. In this review, we analyze the main and well-validated cellular pathways and targets related to senescence as well as their implication in aging-associated diseases. In addition, the most relevant small molecules with senotherapeutic potential, with a special emphasis on their mechanism of action, ongoing clinical trials, and potential limitations, are discussed. Finally, a brief overview of alternative strategies that go beyond the small molecule field, together with our perspectives for the future of the field, is provided.

## 1. Introduction 

Human life expectancy has progressively increased for decades across all developed countries. This demographic trend, however, is not accompanied by the same health and wellness improvement. Hence, one of the hot topics of current biomedicine research is to answer the fundamental question of whether preventing or delaying the manifestation of age-related chronic diseases is possible. This idea means addressing aging with a disease-oriented drug discovery paradigm and, accordingly, identifying altered molecular pathways in the aging process and molecules that can regulate them so that they can generate new drugs.

Within this point of view, aging is considered the main risk factor for many pathological conditions that frequently appear at advanced ages (i.e., >65 years old). These include, among others, immunosenescence, atherosclerosis, hypertension, cardiovascular disorders, type 2 diabetes, sarcopenia, frailty, arthritis, cataracts, deafness, osteoporosis, and neurodegenerative processes such as Alzheimer’s and Parkinson’s diseases, as well as some cancers. Among the accepted hallmarks of aging [1], cellular senescence plays a key role [2,3]. Hence, if signaling pathways and cell functions are altered due to the presence of senescent cells (SnCs), and these cells can be identified and targeted, either by selective killing or rejuvenation approaches, it would be possible at least to slow down the cellular senescence process. It is conceivable that removing aged cells will improve organism and tissue functions and, accordingly, will enable an aged organism to perform as a young one. In this review, we offer a current perspective of the most advanced and well-validated cellular pathways and targets that can be addressed from a drug discovery perspective. We also cover the most important diseases associated with an aged cellular phenotype, the clinical trials currently ongoing, and the development state of small molecules able to halt or reverse these disorders. Finally, newer and more holistic approaches, included in the broad term of geroprotection, are mentioned to finish with our perspectives for the future of this field.

## 2. Cellular Pathways and Targets Involved in Cellular Senescence

**Figure 1:**
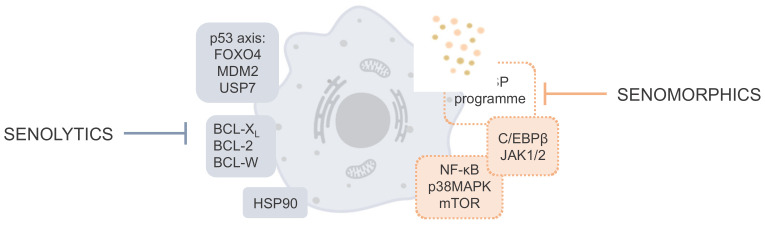
Main signaling pathways and molecular targets for senolytic and senomorphic therapeutic intervention.

Senescence is a cellular state characterized by a stable cell-cycle withdrawal, deregulated metabolism, macromolecular damage, resistance to cell death, and secretion of inflammatory factors known as senescence-associated secretory phenotype (SASP) [4]. Although cellular senescence is an essential physiological program that occurs in normal cells as a response to cellular stress, the long-term presence of SnCs associated with aging has a detrimental role in numerous age-related diseases [2,5]; therefore, targeting cellular senescence by interfering with associated molecular pathways has proved to be a promising opportunity for the prevention and mitigation of aging-related diseases and for increasing life and healthspan —defined as the total years of life lived in good health and without a disability [6,7]. In this sense, diverse therapeutic strategies have emerged, focused on (i) the elimination of SnCs or (ii) the suppression of their detrimental cell-extrinsic effects, including SASP, that are responsible for inflammation and impaired tissue regeneration that drive age-related disorders [8]. This difference allows classifying compounds with anti-senescence potential (globally known as senotherapeutics) into senolytics (compounds able to selectively kill SnCs) and senomorphics (molecules that suppress markers of senescence). In this section, we summarize the main signaling pathways involved in cellular senescence and the proteins that belong to these pathways and can be targeted by senotherapeutic compounds (see Figure 1 for a schematic representation).

Research over the past decade has demonstrated that selective elimination of SnCs extends health and lifespan in animal models [9,10] and can significantly ameliorate aging-associated diseases; therefore, numerous efforts are invested in the development of senolytics that target molecular pathways underlying senescence to selectively kill SnCs. In this sense, resistance to apoptosis is a key characteristic feature of SnCs and inhibition of pro-survival and anti-apoptotic regulators is the most common strategy for the development of senolytics.

Among the different pro-survival pathways within SnCs, the B-cell lymphoma 2 (BCL-2) family of proteins has been widely explored as an attractive target due to its multiple roles in cell fate through the regulation of apoptosis and autophagy [11]. The BCL-2 family can be considered as an apoptotic switch that depends on the interactions between three types of proteins, the pro-survival subfamily and two pro-apoptotic factions, the BH3-only proteins, essential initiators of apoptosis, and the death effector proteins BAX and BAK. The pro-survival cell guardians, BCL-2 itself, BCL-X_L,_ and BCL-W, are upregulated in SnCs and confer resistance to apoptosis-inducing signals [12]. Specifically, these proteins bind to and functionally neutralize the activated pro-apoptotic BAX and BAK proteins, leading to apoptosis inhibition. To date, different specific or dual inhibitors of BCL-2, BCL-X_L,_ and BCL-W proteins have shown senolytic activity in preclinical animal models and in clinical trials (see Table 1 for specific details) by blocking their anti-apoptotic capacity through mitochondrial-mediated mechanisms [12,13,14,15]. Although BCL-2 inhibitors represent the first generation of senolytics, their clinical application is limited by their on-target and dose-limiting toxicity associated with hematological issues, such as neutropenia and thrombocytopenia [16].

The p53 transcription factor axis is another key controller of apoptosis and senescence, making it a promising target for senolytic drug development. p53 is a well-known tumor suppressor and controls a broad range of cellular processes, including cellular stress response, cell cycle arrest, and apoptosis [17]. The levels of p53 undergo an increase in pre-senescent cells after activation of DNA damage response, playing a significant role in the onset of senescence; however, different studies have shown that p53 levels and activity decline in many types of cells after they become senescent, protecting them from apoptosis [18]; therefore, p53 can act as a double-edge sword in senescence, and restoration of its physiological activity can sensitize SnCs and promote cell death by apoptosis. In this sense, the interaction of the forkhead box protein O4 (FOXO4) with p53 plays a key role in the induction of cellular senescence by inhibition of the p53-mediated apoptosis [19]. FOXO4 is highly expressed in SnCs, where it binds and sequesters p53, favoring cell cycle arrest and preventing apoptosis. Thus, interfering with the FOXO4-p53 axis represents a strategy to limit the viability of SnCs, and it has been reported that disrupting the FOXO4 interaction with p53 causes its nuclear exclusion and effectively induces cell-intrinsic apoptosis, ultimately reducing senescence in vitro and in vivo [20].

Activation of p53 is also possible by inhibition of MDM2 (murine double minute 2) protein. This E3 ubiquitin ligase acts as a negative regulator of p53 via proteasome degradation [21], and inhibition of the MDM2/p53 interaction has been shown to restore p53 activity, promoting senescent cell clearance [22]. USP7 (ubiquitin-specific peptidase 7) has been recently described as an alternative approach for p53 upregulation and represents an interesting new senolytic target [23]. This deubiquitinating enzyme protects MDM2 from degradation by the ubiquitin–proteasome system [21], and it has been reported that pharmacological inhibition of USP7 reduces MDM2 expression, which activates p53 and leads to senescent cell apoptosis [24,25,26].

Molecular chaperone HSP90 (heat shock protein 90) is another senolytic target involved in pro-survival pathways in SnCs [27]. HSP90 is implicated in protein folding and stabilization, which makes it essential for the stability of certain anti-apoptotic factors. In fact, SnCs are more dependent on HSP90 than normal cells [28]. Mechanistic studies have revealed that HSP90 protects SnCs against apoptosis via stabilization of AKT or ERK, which are upregulated in senescence. Indeed, targeting HSP90 with small-molecule inhibitors disrupts the interaction with phosphorylated AKT and down-regulates the PI3K/AKT pathway, resulting in selective clearance of SnCs.

Senomorphic or senostatic agents represent an alternative approach to attacking cellular senescence [29,30]; thus, senomorphics are aimed to disrupt the proinflammatory nature of SnCs, keep them alive, or modify their ability to endure cell arrest. The cycle arrest of SnCs is regulated independently of SASP, and this allows us to establish specific senomorphic therapeutic strategies without affecting cell viability.

SASP is mainly composed of growth factors, cytokines, chemokines, and extracellular matrix proteases that affect surrounding cells and reinforce senescence via autocrine or paracrine pathways [29,30]. Its composition varies according to the cell type and senescence cause, but there is a conserved core program that includes proinflammatory interleukins 6 and 8 (IL-6, IL-8) and monocyte chemoattractant protein 1 (MCP-1) [31]. Different signaling pathways are involved in the induction and regulation of SASP, but most of them converge in the activation of the transcription factors NF-κB (nuclear factor-κB) and C/EBPβ (CCAAT/enhancer building protein beta), which orchestrate the SASP production [32]. Interaction of these pathways at different levels could suppress the deleterious pathological effect of SASP and reduce the inflammation associated with aging, thus offering a variety of potential targets for senomorphic intervention.

NF-κB controls cytokine production, transcription of DNA, and cell survival, regulating cellular responses. Reduction in the transcriptional activity of NF-κB, either by direct modulation or by acting on one of its upstream regulators, has been shown to decrease SASP production [33,34]. mTOR (mammalian target of rapamycin) is a serine/threonine protein kinase associated with the NF-κB pathway that was discovered during the mechanistic studies of rapamycin, a well-known senomorphic that defined the potential of mTOR as a senotherapeutic target [35,36]. Thus, blockade of mTOR signaling by rapamycin and other inhibitors results in suppression of the secretion of inflammatory cytokines such as IL-6, and reduces the expression of the upstream regulator of NF-κB activity IL-1α, inhibiting SASP production [36]. mTOR inhibition also affects the MAPK (mitogen-activated protein kinase) pathway, downregulating the MAPKAPK2 translation and ultimately activating NF-κB [35]. Inhibition of other members of the MAPK pathway has also been found to affect NF-κB transcriptional activity and suppress the SASP and its paracrine effects. Specifically, inhibition of p38MAPK limits the secretion of IL-6 and IL-8 cytokines [37,38].

Ataxia telangiectasia mutated (ATM) protein has also emerged as an interesting senomorphic target. This kinase is a key driver of NF-κB-dependent DNA damage-induced senescence, stem cell dysfunction and aging, and it has been reported that its genetic and pharmacological inactivation decreases NF-κB activity, reducing SASP [39]. Another molecular target related to the NF-κB pathway is the silencing information regulator related enzyme 1 (sirtuin 1, SIRT1), an NAD^+^-dependent deacetylase that negatively regulates the nuclear factor signaling. Thus, activation of SIRT1 has been shown to reduce inflammatory cytokine expression by inhibiting NF-κB activity, thereby attenuating cellular senescence [40].

The activity of NF-κB can be directly modulated and it has been shown that direct inhibition can interfere with the translocation of NF-κB to the nucleus, restricting its transcriptional activity and decreasing the capacity of cells to be proinflammatory [41]. NF-κB inhibition, and therefore, SASP attenuation, can be achieved by interaction with IκB kinases (IKK), key regulators in NF-κB activation [42,43].

C/EBPβ is the other critical transcriptional regulator of SASP expression and its activity can be induced by JAK/STAT (Janus kinase/signal transducer and activator of transcription) signaling [32]. This pathway is highly upregulated in SnCs, and there is evidence that genetic and pharmacologic inhibition of JAK1/2 signaling alleviates SASP production [44]. Additionally, the specific action on SASP components could provide a more precise and safer senomorphic strategy. In this sense, the neutralization of IL-1, IL-6, and IL-8 cytokines or their receptors by monoclonal antibodies represents an attractive approach [45].

All these signaling pathways are present in different cells and tissues and their deregulation is behind the appearance of the cellular senescence phenotype, which induces most of the diseases directly related to aging.

## 3. Diseases Related to Senescence

We are living longer than at any point time in human history. The continuous increase in life expectancy is undoubtedly a demographic success that, unfortunately, has not improved hand in hand with healthspan [46]. Conversely, global aging has led to a higher prevalence of chronic age-related pathologies—including cancer, neurodegeneration, chronic pulmonary diseases, cardiovascular diseases, atherosclerosis, diabetes, osteoporosis, osteoarthritis, hepatic dysfunction, renal failure, and blindness—that are responsible for years lived with disability and ultimately are among the major causes of morbidity and death in old age [47]. The World Health Organization (WHO) estimates non-communicable chronic diseases to be the cause of about 41 million deaths yearly, equivalent to 71% of all deaths globally [48]. In this context, the known “geroscience hypothesis” considers aging as the leading risk factor for most serious chronic diseases and disabilities. Thus, addressing an intervention that can slow down the aging process, potentially reducing or postponing the incidence of debilitating age-related diseases, should significantly impact decreasing the enormous social and economic burden caused by chronic diseases [49].

SnCs accumulate with age in different tissues [50], producing the characteristic SASP [31] that contributes to tissue deterioration and ultimately to a variety of diseases and disorders [51]. Beyond cancer, which is considered the aging disease par excellence, herein we discuss the numerous age-related diseases that have been associated with cellular senescence [49,52,53,54,55,56]. Preclinical and clinical studies performed with currently available senotherapeutic drugs are summarized below and in Table 1, whereas the full description of compounds and their mechanisms of action are covered in the following section. Up to this moment, no drug has been approved to treat, delay, or prevent senescence as the main indication since clinical trials are coming along slowly. This is due to the need for a careful risk–benefit balance within first-in-human senolytic clinical trials since potential short- and long-term side effects from clearing SnCs are not yet fully known.

**Table 1:** Senolytic and senomorphic compounds in clinical trials or advanced preclinical studies for age-related diseases.

Compound	Target/Pathway	Clinical Trial Status	
		Age-Related Disease	Registration Number (Phase)
**Senolytics**			
Dasatinib + Quercetin (D + Q)	Numerous (incl. PI3K/AKT and BCL-2)	Alzheimer’s disease	NCT04063124 (1/2)
			NCT05422885 (1/2)
			NCT04685590 (2)
			NCT04785300 (1/2)
		Idiopathic pulmonary fibrosis	NCT02874989 (1)
		Skeletal health	NCT04313634 (2) ^1^
		Chronic kidney disease	NCT02848131 (2)
		Frailty	NCT04733534 (2)
		Diabetic chronic kidney disease	NCT02848131 (2)
		Epigenetic aging	NCT04946383 (2)
		Age-related bone loss	NCT04313634 (2) ^2^
Fisetin	Numerous (incl. PI3K/AKT, BCL-2, p53, and NF-kB)	Frail elderly syndrome	NCT03675724 (2)
			NCT03430037 (2)
			NCT04733534 (2)
		Knee osteoarthritis	NCT04210986 (1/2)
			NCT04770064 (1/2)
			NCT04210986 (1/2)
			NCT04815902 (1/2)
UBX0101	MDM2/p53	Knee osteoarthritis	NCT04129944 (2) ^3^
			NCT04349956 (2)
UBX1325	BCL-X_L_	Age-related macular degeneration	NCT05275205 (2)
			NCT04857996 (2)
Curcumin ^4,5^ and EF-24	Numerous (incl. Nrf2 and NF-kB)	Cellular models of senescence	
Cardiac glycosides (ouabain, digoxin ^5^)	BCL-2, BCL-X_L_ and BCL-W	Preclinical animal models	
ABT-263 ^5^ (Navitoclax)	BCL-2, BCL-X_L_ and BCL-W	Preclinical animal models	
Alvespimycin ^5^ (17-DMAG)	HSP90	Preclinical animal models	
**Senomorphics**			
Rapamycin ^6^	Mtor (also Nrf2 and NF-κB)	Aging	NCT04488601 (2)
			NCT01649960 (1)
			NCT04742777 (2)
			NCT02874924 (2)
			NCT05237687 (2)
		Alzheimer disease	NCT04629495 (2)
		Amyotrophic lateral sclerosis	NCT03359538 (2)
Metformin ^7^	Numerous (incl. IKK, NF-κB, GPx7, and MBNL1)	Aging	NCT03309007 (3)
			NCT02432287 (4)
			NCT04264897 (3)
			NCT03451006 (2)
		Frailty	NCT03107884 (1)
		Muscle atrophy	NCT03107884 (1)
BIRB796 ^8^	p38MAPK	Healthy ^9^	NCT02211885 (1)
			NCT02209805 (1)
RAD001 ^10^	mTOR	Preclinical animal models	
NDGA ^5^	unknown	Preclinical animal models	
SR12343	IKK/NF-κB	Preclinical animal models	
Ruxolitinib ^8,11^	JAK	Preclinical animal models	
SRT12104	SIRT1	Preclinical animal models	

^1^ Fisetin treatment group was also included in the study. ^2^ Treatment includes the concomitant administration of fisetin. ^3^ Not effective. ^4^ Curcumin dietary supplementation is under evaluation (NCT03085680, Phase 2/3) for improving cognitive and physical function in older adults. ^5^ In clinical trials for various cancers. ^6^ Approved for immunosuppression and in more than 1000 clinical trials for other disorders. ^7^ Approved for tuberous sclerosis complex-associated diseases and in more than 500 clinical trials for various cancers. ^8^ In clinical trials for immuno-related disorders. ^9^ Representative studies to assess safety, pharmacokinetics, and pharmacodynamics. ^10^ Approved for type 2 diabetes and in more than 2700 clinical trials for other disorders. ^11^ Approved for graft-versus-host disease.

### 3.1. Neurodegenerative Diseases

Alzheimer’s disease (AD) is nowadays the most common neurodegenerative disease and the most frequent cause of dementia, affecting 50 million people worldwide [57]. Currently approved drugs, e.g., cholinesterase inhibitors and *N*-methyl D-aspartate (NMDA) antagonists, are effective only in treating the symptoms but do not cure or prevent the disease. Parkinson’s disease (PD) is the fastest growing neurological disorder that currently affects people worldwide. Dopamine-based therapies, selective serotonin reuptake inhibitors, and cholinesterase inhibitors help to decrease motor, psychiatric, and cognitive symptoms, respectively; however, no disease-modifying pharmacologic treatments are currently available [58]. In this light, innovative therapeutic approaches are necessary for the treatment of neurodegenerative processes.

Brain functions diminish with age and numerous reports have concluded that cell senescence contributes to the pathogenesis of neurodegenerative diseases [59], especially AD [60] and PD [61], but also of other pathologies such as Huntington’s disease (HD) [62], multiple sclerosis (MS) [63], and amyotrophic lateral sclerosis (ALS) [64]. Neuronal senescence has been demonstrated in the brains of rodents with aging [65], tauopathy [66], and amyloid-β accumulation [67]. Furthermore, a higher SASP activity has been demonstrated in the astrocytes in AD patients [68] and the accumulation of SnCs in the central nervous system has been suggested to contribute significantly to the cognitive decline characteristic of neurological disease. The abundance of SnCs is also associated with deposition of α-synuclein and increased expression of SA-β-gal has been observed in brain tissue from PD patients, suggesting that cell senescence contributes to dopaminergic neurodegeneration [61].

**Figure 2:**
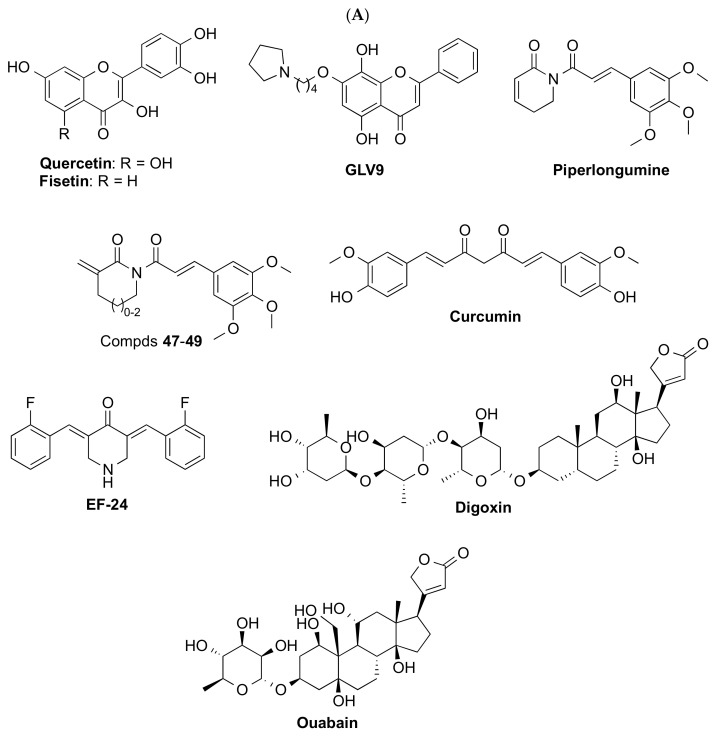

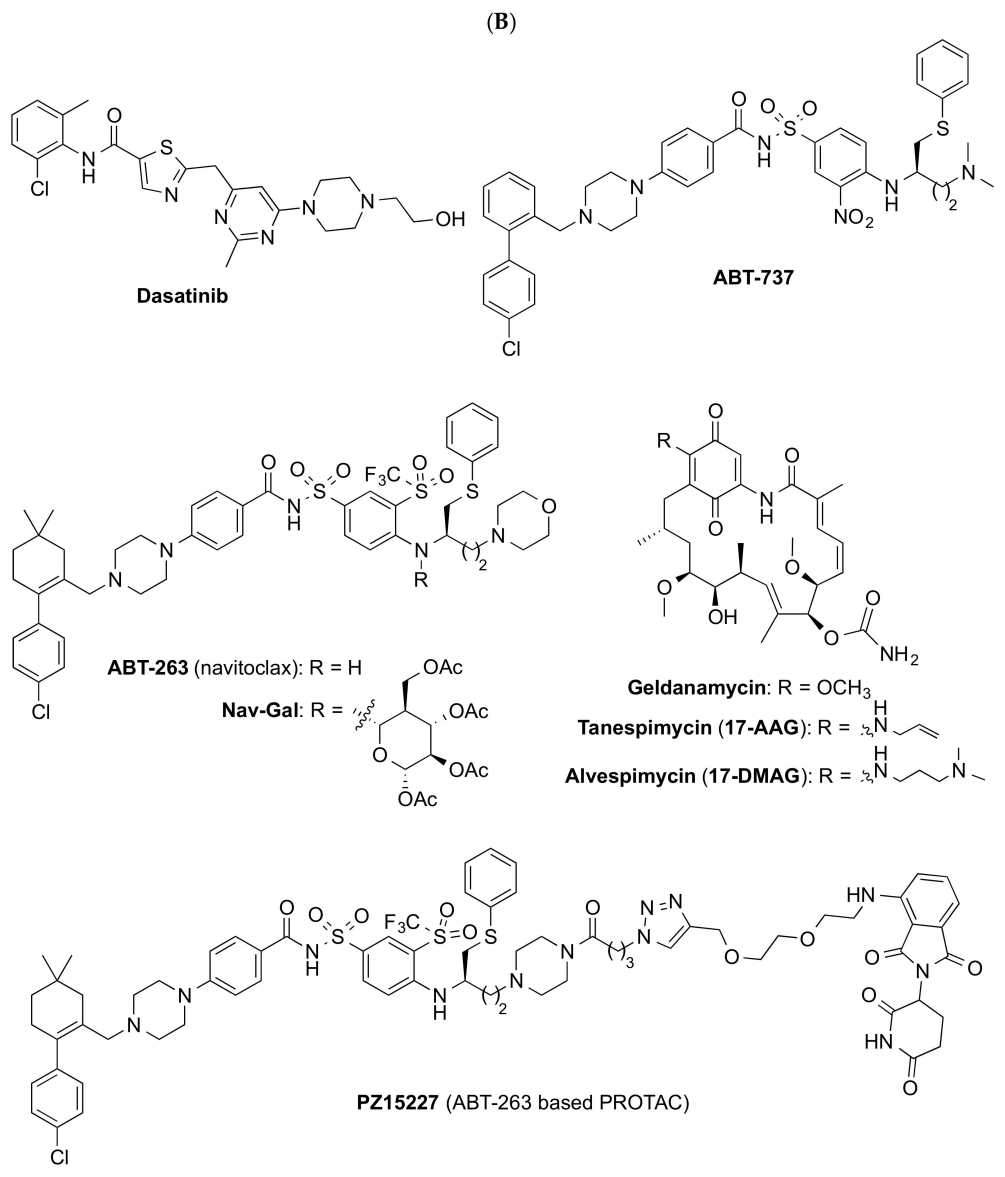
Structure of selected senolytics. (**A**) Natural products. (**B**) Repurposed compounds targeting key enzymes/pathways and representative examples of other approaches based on prodrugs and PROTACs.

**Figure 3:**
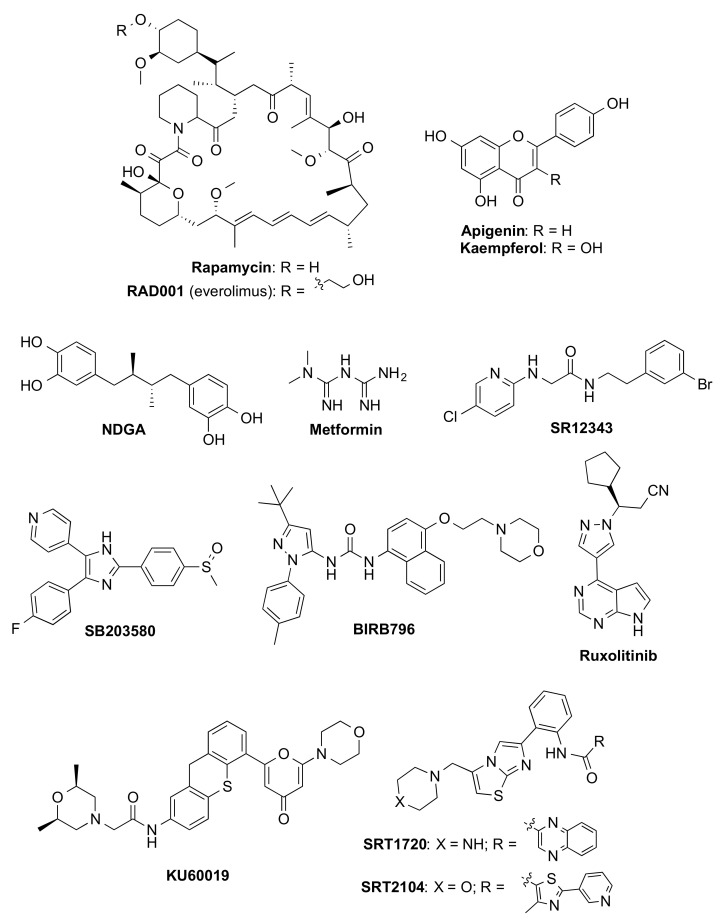
Structure of selected senomorphic compounds.

Notably, clearance or reduction in SnCs by senolytics, e.g., dasatinib and quercetin (D + Q) [67,69], fisetin [70], ABT-263 (navitoclax) [71], and piperlongumine [72], improved relevant outcomes in AD mouse models (see Figure 2 for compound structures). Metformin induced diminished PD pathology in vivo [73]. Senomorphic oral rapamycin [74,75] and metformin [76] (Figure 3) have also been shown to reduce the accumulation of amyloid-β and tau and improve cognition in animal models.

The promising data from preclinical studies and post-mortem human brain tissue have boosted the translation of targeting senescence as an innovative, potentially disease-modifying treatment for AD and PD to the clinic [77]; however, the use of senolytics in humans holds several potential challenges and clinical trials are still in early stages (Table 1 summarizes the most relevant clinical trials currently ongoing with different senotherapeutic compounds). In this regard, D + Q is currently under Phase 2 studies toward amnestic mild cognitive impairment or early AD (NCT04063124, NCT04685590, NCT04785300, and NCT05422885, Table 1). Likewise, Phase 2 clinical trials of senomorphic rapamycin for early AD and ALS (NCT04629495 and NCT03359538, respectively, Table 1) are presently underway.

### 3.2. Respiratory Diseases

Cellular senescence has been hypothesized to play a pathogenic role in two chronic lung pathologies endowed with very limited therapeutic options: idiopathic pulmonary fibrosis (IPF) and chronic obstructive pulmonary disease (COPD) [52,53,78,79,80,81]. Though their full etiology is unknown, some risk factors, such as smoking and aging [82,83], are well established and are suspected to be linked to cellular senescence [84]. In support of this, numerous in vitro studies show evidence of the accumulation of SnCs in lungs from patients with COPD and patients with IPF [85,86].

IPF is a progressive, fatal lung disorder in which abundant fibrotic tissue forms between the alveoli interfering with the gas exchanges. The detrimental role of senescence in lung fibrosis has been supported ex vivo in lung tissue slice cultures from bleomycin-treated mice, in which senescence markers and expression of SASP factors diminished upon the use of senolytics [87]. In vivo, pharmacological or genetic elimination of SnCs also attenuated lung fibrosis and restored lung function in mice models [85,88]. Most importantly, a first-in-human study further showed that senotherapy with D + Q significantly improved pulmonary function during exercise in IPF patients after three weeks of intermittent treatment (NCT02874989, Table 1).

COPD is characterized by progressive airflow limitation and respiratory failure, which is attributed to a combination of small airway fibrosis and emphysema. Treatment with inhaled long-acting bronchodilators improves symptoms and exacerbations but does not reduce disease progression or mortality. In COPD lungs, markers of cellular senescence are evident [89], though it still remains uncertain whether those changes are the cause or consequence with respect to COPD pathogenesis. Nevertheless, targeting cellular senescence has gained increasing attention as a new approach for COPD treatment [90]. Better tolerance to exercise and oxygenation ameliorating the lifespan of animals has been demonstrated using senotherapeutic agents such as rapamycin and its analog (rapalog) everolimus [91], metformin [92], SRT1720 [93] (structures shown in Figure 3), and navitoclax (Figure 2B) [94]. In humans, a study has demonstrated that the use of metformin in diabetic patients with COPD significantly lowers the risk of all-cause mortality [95].

### 3.3. Cardiovascular Diseases

Cardiovascular diseases, principally ischemic heart disease and stroke, are the primary cause of mortality worldwide and a major contributor to disability [96]. These pathologies are often complications of atherosclerosis, a disease characterized by the formation of fibrofatty lesions in the artery wall that begins early in life and progresses gradually, usually remaining asymptomatic for a long period of time but leading to blood flow reduction [97].

Aging leads to cardiac dysfunction that can ultimately cause cardiovascular diseases. Senescence can direct the pathophysiology of these diseases, as different senescent cardiac cell types are known to accumulate upon aging, contributing to cardiac fibrosis and hypertrophy [98,99]. Clinical evidence of the involvement of senescence in atherosclerosis comes from post-mortem histological analysis that showed a senescent cell burden in atherosclerotic substantially higher than in physiologically aged healthy arteries [51,100] and from the development of atherosclerosis in accelerated aging disorders such as the Hutchinson–Gilford progeria syndrome (HGPS) [101,102,103].

Importantly, preclinical studies in genetically modified aged mouse models have demonstrated that clearance of SnCs from cardiovascular organs reverses cardiac fibrosis and hypertrophy [9,49]. Pharmacological decrease in cardiac senescent cell burden upon senolytics (D + Q or navitoclax) treatment resulted in a similar beneficial effect, partly reversing cardiac dysfunction of animal models [98,99,104]. Furthermore, prolonged oral administration of D + Q resulted in a reduction in plaque calcification in naturally aging mice and mice with chronic hypercholesterolemia as a model of atherosclerosis [105]. These studies suggest that senescence inhibition could improve cardiac function and support the emerging role of senolytics as a promising therapeutic option for atherosclerosis and cardiovascular disease management [49,53,54,106,107,108]; however, clinical trials of senolytic therapies in cardiovascular disease are scarce, mainly due to the limited tolerability of toxicity and side effects that limits current senolytic agents.

### 3.4. Diabetes

In low and middle-income countries, diabetes mellitus type 2 (T2D) has risen dramatically and accounts for 1.6 million deaths worldwide each year [109]. Characterized by insulin resistance in peripheral organs, the major risk factors for the development of T2D are obesity and aging, both associated with an increased burden of SnCs. Moreover, diabetic individuals are more likely to develop age-related comorbidities early. Not only is cellular senescence postulated to contribute to the development of T2D, but also diabetes seems to lead to an increased senescent cell accumulation [110]. Hence, the recent identification of senolytics represents an opportunity for testing how senescence is involved in diabetes pathogenesis.

In obese mice, genetic or pharmacological strategies able to promote clearance of SnCs are associated with the improvement of diabetic phenotypes, including glucose tolerance and insulin sensitivity [111]. In obese humans, SnCs accumulate in the adipose tissue, and a senescence signature is found in β cells isolated from T2D patients [112]. Consistently, exposure to senolytics D + Q or fisetin (Figure 2A) in organ cultures of human adipose tissue from patients with diabetes and obesity resulted in a decrease in senescent cell abundance within two days [113,114].

Cellular senescence is also implicated in the pathogenesis of type 1 diabetes (T1D), characterized by insulin deficiency due to the progressive immune-mediated elimination of pancreatic β cells. Elimination of these cells when they are senescent, following the administration of navitoclax, has proven sufficient to protect against T1D development [115,116].

These studies suggest that senotherapeutic interventions might alleviate metabolic dysfunction associated with diabetes. This has been supported in an early, open-label clinical trial of a single three-day course of oral D + Q administration in patients with diabetes complicated by renal dysfunction (NCT02848131, Table 1). As in mice, D + Q successfully reduced senescent cell burden and inflammation in adipose tissue in humans.

Interestingly, synthetic drug metformin (Figure 3), the first-line treatment for T2D, is endowed with senomorphic activity. In humans, a potent effect of the antidiabetic agent on delaying the onset of age-related pathologies has been observed. This prompted the launch of an ongoing clinical study in collaboration with the FDA, aiming at the approval of additional indications for the drug [117].

### 3.5. Musculoskeletal Dysfunctions

Osteoarthritis, a disorder that involves the movable joints, is the leading cause of chronic pain and disability in elderly people [118]. Osteoporosis appears upon aging due to bone loss accompanied by an increasing risk of bone fractures. Aging is also associated with loss of skeletal muscle mass and function, a process defined as sarcopenia, which significantly contributes to frailty and increased mortality in the geriatric population [119]. These age-related musculoskeletal dysfunctions are associated with the accumulation of SnCs in aged cartilage [120], bone [121], or muscle tissues [122]. In the last two decades, the causal role of cellular senescence in these diseases has been demonstrated in preclinical old-mice models by selective clearance of SnCs, achieved by a genetic strategy or senolytic treatment [54]. UBX0101 (structure not disclosed) halts osteoarthritis progression and reduces pain [22]; D + Q leads to increased bone mass and strength [123], as well as improved physical muscle function [113]. In addition, senomorphics rapamycin and metformin promote a regenerative environment in cartilage and exert beneficial effects in osteoarthritic mice [124,125].

Based on preclinical evidence that targeting SnCs may positively affect musculoskeletal system regeneration and age-related pathological progression, a number of clinical trials are currently underway to validate senolytics as a therapy for osteoporosis, osteoarthritis, and sarcopenia [53,126]. UBX0101, via local intra-articular injection, is in Phase 2 studies for knee osteoarthritis (NCT04129944 and NCT04349956, Table 1). Fisetin is also in clinical Phase 2 as an oral treatment for the same pathology (NCT04770064, NCT04210986, and NCT04815902, Table 1). Clinical studies of D + Q and fisetin for age-related bone loss are also in course (NCT04313634). In addition, senomorphic rapamycin is being tested for the aging condition, including bone and muscle loss, in healthy older adults (NCT04488601, Table 1).

### 3.6. Other Diseases

The liver and the kidney undergo age-related alterations in both structure and function that may cause hepatic dysfunction and renal failure, respectively. Empirical evidence from rodent and human studies, such as senescent cell burden or SASP hallmark, points to a role of cellular senescence in the development of chronic hepatic [127,128] and renal diseases [129,130]. In this light, D + Q is under clinical study for chronic kidney disease (NCT02848131, Table 1).

On the other hand, although evidence for a direct role of cellular senescence in ocular diseases remains scarce [131], the senolytic compound UBX1325 (structure not disclosed) is currently in clinical trials for the treatment of age-related macular degeneration and diabetic macular edema (NCT05275205 and NCT04857996, Table 1).

For some reason, the pandemia caused by the severe acute respiratory syndrome coronavirus-2 (SARS-CoV-2), which emerged in 2020, produced a significantly higher mortality rate in chronologically older patients; therefore, the fight against the disease should involve testing the hypothesis that senotherapeutic drugs may have a prominent role in preventing the transmission of the virus, as well as assisting in its treatment [132]. Clinical trials are underway to test whether senolytics, such as fisetin, reduce the progression and morbidity of SARS-CoV-2 in hospitalized older adults [133].

## 4. Senotherapeutic Molecules in Preclinical Studies and Clinical Trials

As previously discussed, the selective elimination of SnCs in mouse models has been demonstrated to extend lifespan and delay the start of age-related pathologies without apparent side effects; therefore, the removal or modulation of SnCs by senotherapeutic drugs has become an attractive approach to prevent, delay, and even revert many of the chronic age-associated disorders and to extend healthspan. As mentioned before, senotherapeutic compounds can be divided into senolytics, which selectively promote the death of SnCs or induce senolysis, and senomorphics that suppress markers of senescence, in particular the SASP, to cause senostasis and prevent the detrimental cell-extrinsic effects of SnCs. This section details the most profoundly characterized small molecules and their mechanism of action in the context of the diseases in which they have been studied.

### 4.1. Senolytics

To date, several classes of senolytic agents have been identified, including natural products and their analogs, compounds derived from the repurposing of anti-cancer drugs targeting critical enzymes involved in pro-survival and anti-apoptotic mechanisms, and other approaches to improve the efficacy or safety (for recent reviews see refs. [7,8,134]). Herein we have summarized the most characterized and advanced compounds of each category that have been validated as senolytics in preclinical models of disease or clinical trials.

#### 4.1.1. Natural Products

Many natural products display anti-oxidant and anti-inflammatory activities, so it is expected that some of them show anti-aging effects. In fact, several natural products have been used as traditional medicines and nutritional supplements to prevent or treat age-related diseases; however, only a few of them have been properly identified as senolytics, although their mechanisms of action have not always been well defined. Among them, compounds such as the flavonoids quercetin and fisetin, piperlongumine, curcumin, and cardiac glycosides deserve special attention (Figure 2A).

Quercetin is a dietary flavonoid with diverse biological activities [135], including interacting with a PI3K isoform and BCL-2 family members (Table 1), which has been used as a nutritional supplement and phytochemical treatment for diabetes, obesity, cardiovascular dysfunction, inflammation, and mood disorders. This flavonoid, with strong anti-oxidant activity, was characterized as a moderate senolytic in 2015, being capable of clearing only some specific types of cells, such as endothelial cells, but not senescent preadipocytes [136]. Interestingly, its combination with dasatinib, a tyrosine kinase inhibitor approved by the FDA as an anti-cancer drug, induces apoptosis more efficiently by targeting more SnC anti-apoptotic pathways (SCAPs) than either drug alone [136]. Moreover, the combination treatment D + Q delayed many age-related diseases, extending the healthspan in mice, and has demonstrated its efficacy in mouse models of atherosclerosis [105], pulmonary fibrosis [85], hepatic steatosis [137], AD [67], and obesity [138] among others. This senolytic combination has entered several clinical trials, as summarized in Table 1.

Fisetin is another flavonoid found in a variety of fruits and vegetables that has shown beneficial biological effects such as anti-oxidant, anti-cancer, anti-inflamatory, anti-diabetic, antiviral, and neuroprotective activities [139]. This flavonoid exerts these effects through diverse mechanisms of action on multiple molecular targets and signaling pathways, including BCL-2, PI3K/AKT, and p53 [140] (Table 1). In 2017, fisetin was first characterized as a senolytic compound able to selectively kill SnCs [14]. Treatment of progeroid *Erccl*^−/Δ^ or naturally aged mice with fisetin reduced SnC burden in multiple tissues, improving tissue homeostasis, reducing age-related pathologies, and extending lifespan [114]. The human efficacy of this flavonoid in age-related diseases is currently being evaluated in several clinical trials, as shown in Table 1.

GL-V9, a synthetic flavonoid derivative of wogonin, has shown senolytic activity in senescent breast cancer cells, by inducing ROS-dependent apoptosis [141], and in malignant T-cell lines [142], although further studies are required to elucidate its mechanism of action.

Piperlongumine is a natural amide alkaloid isolated from long pepper, which exerts senolytic effects in senescent WI38 fibroblasts [143]. Although its precise senolytic mechanism of action is still unclear, it has been demonstrated that piperlongumine selectively kills SnCs by directly binding to oxidation resistance 1 (OXR1), leading to its proteasomal degradation and increasing ROS production [144]. A series of structural modifications around piperlongumine afforded analog compounds 47–49 (Figure 2A) with improved senolytic activity [145].

Curcumin, a hydrophobic polyphenol isolated from the rhizome of *Curcuma longa*, is recognized and used worldwide in many different forms for multiple potential health benefits [146]. This natural product has been shown to clear human senescent intervertebral disc cells by down-regulating the Nrf2 and NF-κB pathways [147] (Table 1). Its synthetic analog EF-24 (Figure 2A), with improved bioavailability, displays more potent senolytic activity in several SnCs by inducing cellular apoptosis through an increase in the proteasome degradation of the BCL-2 anti-apoptotic protein family [148] (Table 1).

Cardiac glycosides ouabain and digoxin (Figure 2A), secondary metabolites found in several plants, have been recently characterized as senolytic compounds by high throughput screening [149,150]. These compounds caused the death of several types of SnCs from different species and tissues origins probably by targeting the Na^+^/K^+^ ATPase pump, thus causing an imbalanced electrochemical gradient within the cell, which produced depolarization and acidification. Ouabain and digoxin activate the gene expression of the pro-apoptotic BCL2-family, mainly the protein NOXA [150] (Table 1). They also exhibit strong senolytic activity in several mouse models of age-related diseases, such as lung fibrosis [149], which supports their potential as a therapeutic treatment of age-associated pathologies.

#### 4.1.2. Repurposed Compounds

Targeted senolytics identified to date are mainly repurposed anti-cancer drugs that target SCAPs (Figure 2B). These compounds are, in general, more potent senolytics than natural products, with the exception of cardiac glycosides. Nonetheless, repurposed senolytics usually display many on-target and/or off-target toxicities, which can hamper their clinical translation as anti-aging drugs; therefore, new strategies to develop safer targeted senolytics are required.

**Inhibitors of the BCL-2 family proteins.** Some BCL-2 inhibitors, such as ABT-737 and ABT-263 (Figure 2B), have been identified as a novel class of senolytics. ABT-737 is able to eliminate SnCs in mice in the lung and epidermis [12], although it has poor solubility and is not orally bioavailable. Structural modifications led to its derivative ABT-263 (navitoclax, Table 1), an orally bioavailable pan-BCL inhibitor [151], which can selectively clear SnCs in various murine tissues and ameliorates pathological conditions associated with aging, such as dementia [152], atherosclerosis [13], and pulmonary fibrosis [71]. Navitoclax has reached clinical trials for several types of cancer [7]; however, its inhibition of other members of the family, such as BCL-X_L_, produces thrombocytopenia, which impedes its clinical translation for age-related diseases; therefore, new strategies to overcome the on-target toxicity of navitoclax and to advance the BCL-2 inhibitors to clinical translation are under study.

**HSP90 inhibitors**. Several HSP90 inhibitors, such as geldanamycin, tanespimycin (17-AAG), and alvespimycin (17-DMAG) (Figure 2B), have been identified as senolytics able to kill a variety of SnCs in mouse and human [27] and the treatment of *Erccl*^−/Δ^ progeroid mice with 17-DMAG delayed the onset of several age-related phenotypes and diseases [153] (Table 1); however, 17-DMAG displays poor pharmacokinetic and pharmacodynamic properties, which has boosted the search for new analogs with safer profile to reach HSP90 inhibitors into the clinic for the treatment of age-related pathologies.

**p53 pathway targeting compounds**. The increase in p53 transcriptional activity via disruption of its interaction with FOXO4 or MDM2 has been hypothesized as a senescence pathway. The inhibition of FOXO4/p53, with the designed peptide FOXO4-DRI [20], or MDM2/p53, with the compound UBX0101 (structure not disclosed) [22], effectively cleared SnCs in mice. In fact, local treatment with UBX0101 selectively kills SnCs in mice with post-traumatic osteoarthritis and Unity Biotechnology advanced this compound to clinical trials for the treatment of this pathology (Table 1); however, the Phase 2 clinical trial for osteoarthritis did not result in statistical significance compared with the placebo; therefore, the development of more efficient and safer approaches to activate p53 without causing significant tissue toxicity is still required.

#### 4.1.3. Other Senolytic Approaches

Several approaches to increase the specificity and safety profile of the senolytic small molecules identified, mainly based on the use of drug delivery approaches, are currently under development [154]. Based on the fact that most of the reported SnC types share an elevated activity of the lysosomal senescence-associated β-galactosidase activity (SA-β-gal), pro-drugs with a cleavable galactose moiety attached to a senolytic compound can lead to the specific release of the compound of interest in SnCs. This strategy has been successfully applied to the galactose-modified BCL inhibitor navitoclax, in which the attachment of peracetylated galactose led to the pro-drug Nav-Gal (Figure 2B) with increased senolytic specificity and lower platelet toxicity than navitoclax [155]. Based on a similar approach, nanoparticles containing cytotoxic compounds coated with galacto-oligosaccharides on a silica scaffold have been employed to selectively deliver cytotoxic compounds to SnCs [5]. Thus, treatment with navitoclax encapsulated in galactose nanoparticles increased the efficacy of the drug in reducing tumor growth in a mouse model of triple-negative breast cancer [156]. Nonetheless, the efficacy and safety of these drug delivery strategies have not been validated yet for the treatment of other senescence-induced diseases.

The reduction in the platelet toxicity of navitoclax has also been accomplished using a proteolysis targeting chimera (PROTAC) methodology by tethering this compound to a pomalidomide moiety through a linker. The obtained derivative PZ15227 (Figure 2B) is a selective BCL-X_L_ PROTAC, which targets this protein to the cereblon (CRBN) E3 ligase for degradation [157]. Compared to navitoclax, PZ15227 is slightly more potent against SnCs but less toxic to platelets since CRBN is poorly expressed in platelets [20]. The in vivo efficacy of PZ15227 has been demonstrated in naturally aged mice; therefore, BCL-X_L_ PROTACs might become safer and more potent senolytics than BCL-X_L_ inhibitors, although pharmacokinetic and pharmacodynamic studies are required to ensure the adequate absorption of PZ15227 due to its high molecular weight.

### 4.2. Senomorphics

Most of the senomorphic agents identified so far (Figure 3) have been discovered by serendipity, such as rapamycin and metformin. Some of them are natural products (i.e., apigenin and kaempferol), which act as free radical scavengers or are able to modulate the detrimental effects of the SnCs via the inhibition of SASP components through multiple mechanisms. Others are synthetic compounds that can be divided into inhibitors of NF-κB, p38MAPK, JAK pathways, and ATM, or sirtuin-activating compounds (STACs).

**Synthetic and natural products**. Rapamycin (Figure 3) is a macrolide isolated from *Streptomyces hygroscopicus* with antifungal properties [158], approved by the FDA to prevent organ rejection in kidney transplantation and for the treatment of lymphangioleiomyomatosis. Several studies have revealed that this macrolide and its analog RAD001 (everolimus, Figure 3) reduce cellular senescence, suppressing SASP and extending not only lifespan but also healthspan by slowing or even reversing age-related changes in mice, including heart dysfunction [159] and cognitive deficits [160]. The senomorphic effects of rapamycin are mainly related to its inhibition of the mTOR signaling pathway [161], although other secondary mechanisms, such as the activation of the Nrf2 pathway [162] or decreasing NF-κB activity [36], seem to be involved (Table 1). Although rapamycin is the more deeply characterized senomorphic compound, its clinical translation for the treatment of age-related pathologies is still under evaluation (Table 1) and might be limited by its toxic side effects, which include hyperglycemia, hyperlipidemia, thrombocytopenia, kidney toxicity, and immunosuppression probably due to its off-target inhibition of mTORC2 [163]; therefore, the development of new rapamycin analogs able to selectively reduce mTORC1 signaling may be an interesting approach to extend the healthspan.

Several natural products, such as the flavonoids apigenin and kaempferol or nordihydroguaiaretic acid (NDGA) (Figure 3), have demonstrated their senomorphic activity by inhibiting SASP production in bleomycin-induced senescence fibroblasts [164], or in senescent *Erccl*^–/Δ^ mice [165], respectively, although their mechanisms of action are still unclear.

Metformin (Figure 3), a synthetic biguanide, approved for the treatment of T2D for more than 60 years, is effective in suppressing cellular senescence and SASP in different types of SnCs, attenuating multiple age-related dysfunctions in animal models through multiple pathways, such as IKK, NF-κB, GPx7, and MBNL1, in a complex manner. Due to the geroprotective action of the treatment with metformin in diabetic patients [166,167], safety and low cost, this compound will be tested in the Targeting Aging with Metformin (TAME) initiative to study its effect on 3000 non-diabetic individuals, aged 65–79 years, in multicenter six-year clinical trials in the United States [168] and is currently under evaluation in several clinical trials related to aging, frailty and muscle atrophy (Table 1).

**NF-κB inhibitors**. Pharmacological inhibition of the transcription factor NF-κB with a peptide inhibitor of IKK, termed the NEMO-binding domain (NBD), delays the onset of progeroid symptoms in *Erccl*^–/Δ^ mice [169]. To overcome the poor pharmacokinetic profile of this peptide, a virtual screening followed by structural optimization led to the identification of the small molecule SR12343 (Figure 3) as an IKK/NF-κB inhibitor that reduces senescence and SASPs in vitro and extends healthspan in vivo in naturally aged mice and in several models of accelerated aging [170].

**p38MAPK inhibitors**. Several p38MAPK inhibitors, such as SB203580 [37] and BIRB796 [171] (Figure 3), reduce the SASP secretion, although a better understanding of the signaling pathways underlying the SASP is still required. In fact, BIRB796, which is a more potent suppressor of SASP and a more selective p38MAPK inhibitor than SB203580, was used to demonstrate that SASP secretion is p38-dependent in human fibroblasts [171]. BIRB796 has reached several clinical trials to evaluate its safety profile and efficacy for the treatment of inflammatory diseases (Table 1), although its clinical evaluation for age-related pathologies has not been assessed yet.

**JAK/STAT inhibitors**. The JAK/STAT pathway is more up-regulated in senescent than in non-SnCs. Inhibition of the JAK pathway by the JAK 1/2 inhibitor ruxolitinib (Figure 3) suppressed SASP production [44], alleviated age-related dysfunction in several mouse models [44,172], and reduced frailty in old age.

**ATM inhibitors**. ATM is a key protein kinase that is persistently elevated in *Erccl*^−/Δ^ progeroid and naturally aged mice [39]. Its inhibition with KU-600193 (structure shown in Figure 3) alleviated cellular senescence by recovering mitochondrial function in normal fibroblasts [173] and in accelerated aging cells [174]; however, ATM activity has to be finely tuned to achieve beneficial effects in reducing senescence, while minimizing its potential risk to generate cancer due to the important role of this protein in DNA repair.

**STACs**. SIRT1 enzyme regulates many signaling and transcriptional pathways involved in senescence and aging [40]. Activation of SIRT1 by resveratrol prevents cellular senescence and suppresses SASP in several cell types [175,176]. To improve the bioavailability and stability of resveratrol, other STACs, such as SRT1720 and SRT2104 (Figure 3), have been developed, extending the lifespan of mice [177]. Among them, SRT2104 deserves special attention due to its good bioavailability in humans and has entered several clinical trials for the treatment of age-related disorders (Table 1). 

## 5. Other Approaches

Other approaches for confronting mechanisms of aging focused on increasing healthspan have been described in the last years. Although they are in a less developed state from the point of view of drug discovery, they will probably play an important role in the next years. Among them, the main lines of research include the impact of the blood circulating factors, the influence of the microbiome, strategies focused on the rejuvenation of the immune system, and holistic approaches encompassed in the general term of geroprotection, which include lifestyle aspects such as nutrition, exercise, and calorie restriction. Although these approaches are still in a very early maturation state with respect to their realistic clinical application, the underlying aim is that this systematic research can originate new drugs in the future able to significantly extend the current healthspan.

### 5.1. Blood Circulating Factors

Aging is characterized by a general impaired ability for tissue regeneration. Hence, the question that emerges is whether this feature is intrinsic to the cellular state or is somehow influenced by the environment. Heterochronic parabiosis experiments, in which two individuals of different ages shared a circulatory system, were designed to address this subject [178,179]. Initial observations suggested that young blood could improve the age-related effects if inoculated in an old mouse by improving aged skeletal muscle stem cell and hepatocyte proliferation [180]. Similarly, young blood can reverse the age-related deleterious effects in remyelination [181], neurogenesis and cognitive function [182,183], kidney deterioration [184], decline in pancreatic β-cell replication [185], and in bone repair [186] and vascular dysfunction [187,188]. Collectively, these results suggest two important concepts: (i) that there are systemic blood factors that change with age, and (ii) that these factors can modulate, halt, or even reverse tissue senescence. Considering the technical difficulties associated with regular plasma infusion, youth plasma availability, development of well-designed clinical trials, eventual agency approval, and the ethical concerns that could arise, the logical quest is to identify those factors and signaling pathways. In this way, the former could provide new drugs and the latter novel pharmacological approaches to dissociate physiological aging from tissue deterioration.

The advent of new -omics and single-cell analysis technologies has allowed us to start to delineate the specific (macro)molecules and the mechanisms of action involved in pro- or anti-senescent effects. These factors include chemokines such as CCL11 [182] or the β2-microglobulin (B2M) protein [189], which, at increased levels, impair neurogenesis, learning, and memory. Other pro-aging factors include the actin-associated protein tropomyosin 1 (TPM1) [190]. On the contrary, sustained levels of tissue inhibitor of metalloproteinases 2 (TIMP2) have been involved in hippocampal-dependent cognition enhancement [191] and fibroblast growth factor 17 (Fgf17) infusion is sufficient to induce the proliferation of oligodendrocyte progenitor cells (OPC) and long-term memory consolidation in aged mice. Conversely, Fgf17 blockade impairs cognition in young mice. These findings pinpoint Fgf17 as a key mediator in preserving the oligodendrocyte function in the aging brain [192]. In relationship to signaling pathways, activation of the cyclic AMP response element binding protein (Creb) induced structural and cognitive enhancements in the aged hippocampus [183], whereas inhibition of the canonical β-catenin pathway during early stages of injury improves bone healing in aged mice [186].

Among the blood circulating factors, cells are a key part of them, as whole blood contains not only ions and small macromolecules but also many different cell types, including erythrocytes and immune system cells. In this regard, an aged hematopoietic system has been linked to hippocampal-dependent cognitive deterioration. Among the specific factors responsible for this effect, cyclophilin A protein has been identified as a pro-aging factor [193]. It is interesting to note that while, as indicated before, young blood can restore many aged tissues, it cannot rejuvenate itself, i.e., aged hematopoietic stem cells (HSCs) remain unaffected by systemic anti-aging strategies such as parabiosis, exercise, or calorie restriction [194].

### 5.2. The Gut Microbiome

The gut microbiome is emerging as a key regulator of several metabolic, immune, and neuroendocrine pathways [195,196]. Given its implications for many conditions such as obesity, type 2 diabetes, cardiovascular disease, non-alcoholic fatty acid liver disease, and cancer [196,197,198,199], its role in aging has also received attention [200]. In this regard, different studies have addressed the characterization of the gut microbiome in normal aging [201,202] and in accelerated aging diseases such as HGPS [203], aimed at identifying fundamental differences that could be therapeutically exploited in the form of specific pre- o pro-biotic administration. In this context, diverse studies have suggested that the longevity of centenarians is positively related to the abundance of beneficial commensals such as *Akkermansia muciniphila* [202,203,204,205]. Subsequent metabolomics analyses carried out in independent studies have pointed to the importance of secondary bile acids as signaling key mediators in the observed beneficial effects [203] and specifically have suggested the importance of isoallolithocholic acid [205]. Collectively, these results support the existence of a link between aging and the gut microbiome and provide a rationale for microbiome-based interventions against age-related diseases.

### 5.3. Immune System

Aging is a multifactorial phenomenon that affects basically all organ systems and cellular processes, with the immune system being one of the most altered [206]. Almost all types of immune cells vary with age in terms of numbers and/or activity; however, these alterations are in general highly detrimental, leading to higher susceptibility to infections, reduced healing capacity, and altered homeostasis that favor the development of age-associated diseases such as cancer, diabetes, and other pathologies associated with inflammation; thus, significantly affecting the overall well-being of the organism [1]. One example we have just witnessed is how aging has been defined as a strong risk factor for disease severity and mortality upon infection with the SARS-CoV-2 [207]. This fact is closely related to the immune dysfunction that characterizes the elderly that precludes a robust immune response. The dysfunctional immune system in aging has been associated with two processes defined as “immunosenescence” and “inflammaging”. The former refers to the gradual deterioration of the immune system that involves a loss in its capacity to respond to infections and to generate an effective, long-lasting immune memory. The latter describes the situation in which immune cells such as HSCs, microglia, granulocytes, and T lymphocytes are characterized by a chronic and increased production of inflammatory cytokines that characterizes the senescence-like state. Different anti-aging therapeutic approaches targeting specific immune dysfunctions in the elderly are being proposed at a growing pace, with many of them displaying potential in preclinical studies. For example, the presence of specific protein markers that identify senescent T cells could open the possibility for therapies targeting these specific populations without affecting normal immune system functions. In this sense, a CD153 peptide vaccine that uses a specific peptide to induce high production of anti-CD153 antibodies has been recently described [208]. Another possibility relies on strategies aimed at inducing telomere elongation [209] since T cells from healthy centenarians show longer telomeres and higher telomerase activity in response to stimulation compared to other centenarians.

### 5.4. Geroprotection

The term geroprotection comprises all the strategies aimed at the slowdown, inhibition, or reversal of age-related decline. It implies changes in lifestyle directed to preserve the individual’s independence, physical function, and cognition, taking advantage of integral organism interventions that include nutrition with special attention to the consumption of anti-oxidants and other smart foods, calorie restriction regimens, and exercise [210,211]. Among the different interventional strategies, exercise has received special attention and has been systematically studied. In particular, exercise has been described to reverse age-related declines in adult neurogenesis and cognitive function in the aged hippocampus [212,213], which is a brain region sensitive to the detrimental effects of aging. Hence, identification of the molecular factors responsible for the beneficial effects of exercise is of critical importance to designing therapeutic approaches, especially considering that, in the elderly, the eagerness or the capacity for performing physical exercise on a routine basis can be limited by physical frailty or poor health. With this idea in mind, recent studies have confirmed that administration of circulating blood factors in plasma from exercised aged mice transferred the effects of exercise on adult neurogenesis and cognition to sedentary aged mice. To identify the individual circulating blood factors that mediated these effects, liquid chromatography coupled with tandem mass spectrometry was carried out. Determination of the relative amounts of soluble proteins in the plasma from exercised or sedentary aged and mature mice characterized the enzyme glycosylphosphatidylinositol (GPI) specific phospholipase D1 (Gpld1), as an exercise-induced circulating blood factor in aged mice and humans with potential relevance to cognitive function in mice. The effect of Gpld1 seemed to be mediated by altering signaling cascades downstream of GPI-anchored substrate cleavage. These findings imply a liver-to-brain axis by which blood factors can transfer the benefits of exercise in old age [214].

## 6. Conclusions and Future Perspectives

Aging is commonly regarded as an inevitable part of the life cycle; however, current research suggests that it may not be the inexorable process we consider it at the present moment. Actually, obtained results with different models indicate that (i) cells become senescent as time passes; (ii) SnCs have altered functions, which eventually lead to aging-related diseases; (iii) aged cells are different from young cells and these differences can be exploited for specific targeting; (iv) senescent cell removal or rejuvenation strategies involve improvements in aging-related pathological states; (v) there exist compounds (that may become drugs in the near future) that, by correcting and modulating cellular senesce can slow down, halt or even reverse aging-related diseases. Globally, these results suggest that aging is a druggable process that can be targeted with the appropriate drugs, similar to other chronic disorders. In this context, and as detailed in this review, different compounds are being actively assayed and their mechanism(s) of action characterized. Although their potential, specific clinical indication, and long-term safety remain to be confirmed in clinical trials, current evidence suggests that the ongoing approaches may revolutionize the longevity field; however, current limitations include the difficulty of performing well-designed clinical trials, as usually elderly patients are typically multimorbid and take simultaneously several drugs. Hence, findings from clinical trials performed with elderly patients may be affected by drug–drug interactions, complicating the evaluation of effects (either beneficial or harmful) of studied drugs. In addition, another important challenge in designing such clinical trials is to select the appropriate outcome measures. In addition, a better understanding of the cellular and molecular pathways that underlie the senescence and the rejuvenation processes is still required, since this biological knowledge would allow us to explore not only the known but possibly new therapeutic approaches for achieving the long-sought goal of healthspan extension. Consecution of this global and ambitious aim can change the paradigm of life as we conceive it today, and will have fundamental implications for society and health systems as a whole.

## References

[1] López-Otín C., Blasco M.A., Partridge L., Serrano M., Kroemer G. (2013). The hallmarks of aging. Cell.

[2] Van Deursen J.M. (2014). The role of senescent cells in ageing. Nature.

[3] Gerdes E.O.W., Zhu Y., Tchkonia T., Kirkland J.L. (2020). Discovery, development, and future application of senolytics: Theories and predictions. FEBS J..

[4] Hayflick L., Moorhead P.S. (1961). The serial cultivation of human diploid cell strains. Exp. Cell Res..

[5] Muñoz-Espín D., Rovira M., Galiana I., Giménez C., Lozano-Torres B., Paez-Ribes M., Llanos S., Chaib S., Muñoz-Martín M., Ucero A.C. (2018). A versatile drug delivery system targeting senescent cells. EMBO Mol. Med..

[6] Childs B.G., Gluscevic M., Baker D.J., Laberge R.-M., Marquess D., Dananberg J., van Deursen J.M. (2017). Senescent cells: An emerging target for diseases of ageing. Nat. Rev. Drug Discov..

[7] Di Micco R., Krizhanovsky V., Baker D., d’Adda di Fagagna F. (2021). Cellular senescence in ageing: From mechanisms to therapeutic opportunities. Nat. Rev. Mol. Cell Biol..

[8] Zhang L., Pitcher L.E., Prahalad V., Niedernhofer L.J., Robbins P.D. (2022). Targeting cellular senescence with senotherapeutics: Senolytics and senomorphics. FEBS J..

[9] Baker D.J., Childs B.G., Durik M., Wijers M.E., Sieben C.J., Zhong J., Saltness R.A., Jeganathan K.B., Verzosa G.C., Pezeshki A. (2016). Naturally occurring p16Ink4a-positive cells shorten healthy lifespan. Nature.

[10] Baker D.J., Wijshake T., Tchkonia T., LeBrasseur N.K., Childs B.G., van de Sluis B., Kirkland J.L., van Deursen J.M. (2011). Clearance of p16Ink4a-positive senescent cells delays ageing-associated disorders. Nature.

[11] Czabotar P.E., Lessene G., Strasser A., Adams J.M. (2014). Control of apoptosis by the BCL-2 protein family: Implications for physiology and therapy. Nat. Rev. Mol. Cell Biol..

[12] Yosef R., Pilpel N., Tokarsky-Amiel R., Biran A., Ovadya Y., Cohen S., Vadai E., Dassa L., Shahar E., Condiotti R. (2016). Directed elimination of senescent cells by inhibition of BCL-W and BCL-XL. Nat. Commun..

[13] Chang J., Wang Y., Shao L., Laberge R.-M., Demaria M., Campisi J., Janakiraman K., Sharpless N.E., Ding S., Feng W. (2016). Clearance of senescent cells by ABT263 rejuvenates aged hematopoietic stem cells in mice. Nat. Med..

[14] Zhu Y., Doornebal E.J., Pirtskhalava T., Giorgadze N., Wentworth M., Fuhrmann-Stroissnigg H., Niedernhofer L.J., Robbins P.D., Tchkonia T., Kirkland J.L. (2017). New agents that target senescent cells: The flavone, fisetin, and the BCL-XL inhibitors, A1331852 and A1155463. Aging.

[15] Zhu Y., Tchkonia T., Fuhrmann-Stroissnigg H., Dai H.M., Ling Y.Y., Stout M.B., Pirtskhalava T., Giorgadze N., Johnson K.O., Giles C.B. (2016). Identification of a novel senolytic agent, navitoclax, targeting the Bcl-2 family of anti-apoptotic factors. Aging Cell.

[16] Cang S., Iragavarapu C., Savooji J., Song Y., Liu D. (2015). ABT-199 (venetoclax) and BCL-2 inhibitors in clinical development. J. Hematol. Oncol..

[17] Vousden K.H., Prives C. (2009). Blinded by the light: The growing complexity of p53. Cell.

[18] Rufini A., Tucci P., Celardo I., Melino G. (2013). Senescence and aging: The critical roles of p53. Oncogene.

[19] Bourgeois B., Madl T. (2018). Regulation of cellular senescence via the FOXO4-p53 axis. FEBS Lett..

[20] Baar M.P., Brandt R.M.C., Putavet D.A., Klein J.D.D., Derks K.W.J., Bourgeois B.R.M., Stryeck S., Rijksen Y., van Willigenburg H., Feijtel D.A. (2017). Targeted apoptosis of senescent cells restores tissue homeostasis in response to chemotoxicity and aging. Cell.

[21] Kruse J.P., Gu W. (2009). Modes of p53 regulation. Cell.

[22] Jeon O.H., Kim C., Laberge R.-M., Demaria M., Rathod S., Vasserot A.P., Chung J.W., Kim D.H., Poon Y., David N. (2017). Local clearance of senescent cells attenuates the development of post-traumatic osteoarthritis and creates a pro-regenerative environment. Nat. Med..

[23] He Y., Li W., Lv D., Zhang X., Zhang X., Ortiz Y.T., Budamagunta V., Campisi J., Zheng G., Zhou D. (2020). Inhibition of USP7 activity selectively eliminates senescent cells in part via restoration of p53 activity. Aging Cell.

[24] Chauhan D., Tian Z., Nicholson B., Kumar K.G., Zhou B., Carrasco R., McDermott J.L., Leach C.A., Fulcinniti M., Kodrasov M.P. (2012). A small molecule inhibitor of ubiquitin-specific protease-7 induces apoptosis in multiple myeloma cells and overcomes bortezomib resistance. Cancer Cell.

[25] Fan Y.H., Cheng J., Vasudevan S.A., Dou J., Zhang H., Patel R.H., Ma I.T., Rojas Y., Zhao Y., Yu Y. (2013). USP7 inhibitor P22077 inhibits neuroblastoma growth via inducing p53-mediated apoptosis. Cell Death Dis..

[26] Tavana O., Li D., Dai C., Lopez G., Banerjee D., Kon N., Chen C., Califano A., Yamashiro D.J., Sun H. (2016). HAUSP deubiquitinates and stabilizes N-Myc in neuroblastoma. Nat. Med..

[27] Fuhrmann-Stroissnigg H., Ling Y.Y., Zhao J., McGowan S.J., Zhu Y., Brooks R.W., Grassi D., Gregg S.Q., Stripay J.L., Dorronsoro A. (2017). Identification of HSP90 inhibitors as a novel class of senolytics. Nat. Commun..

[28] Pluquet O., Pourtier A., Abbadie C. (2015). The unfolded protein response and cellular senescence. A review in the theme: Cellular mechanisms of endoplasmic reticulum stress signaling in health and disease. Am. J. Physiol. Cell Physiol..

[29] Acosta J.C., Banito A., Wuestefeld T., Georgilis A., Janich P., Morton J.P., Athineos D., Kang T.W., Lasitschka F., Andrulis M. (2013). A complex secretory program orchestrated by the inflammasome controls paracrine senescence. Nat. Cell Biol..

[30] Coppe J.P., Desprez P.Y., Krtolica A., Campisi J. (2010). The senescence-associated secretory phenotype: The dark side of tumor suppression. Annu. Rev. Pathol..

[31] Coppe J.P., Patil C.K., Rodier F., Sun Y., Munoz D.P., Goldstein J., Nelson P.S., Desprez P.Y., Campisi J. (2008). Senescence-associated secretory phenotypes reveal cell-nonautonomous functions of oncogenic RAS and the p53 tumor suppressor. PLoS Biol..

[32] Faget D.V., Ren Q., Stewart S.A. (2019). Unmasking senescence: Context-dependent effects of SASP in cancer. Nat. Rev. Cancer.

[33] Osorio F.G., Soria-Valles C., Santiago-Fernandez O., Freije J.M., Lopez-Otin C. (2016). NF-kappaB signaling as a driver of ageing. Int. Rev. Cell Mol. Biol..

[34] Salminen A., Kauppinen A., Kaarniranta K. (2012). Emerging role of NF-kappaB signaling in the induction of senescence-associated secretory phenotype (SASP). Cell Signal..

[35] Herranz N., Gallage S., Mellone M., Wuestefeld T., Klotz S., Hanley C.J., Raguz S., Acosta J.C., Innes A.J., Banito A. (2015). mTOR regulates MAPKAPK2 translation to control the senescence-associated secretory phenotype. Nat. Cell Biol..

[36] Laberge R.-M., Sun Y., Orjalo A.V., Patil C.K., Freund A., Zhou L., Curran S.C., Davalos A.R., Wilson-Edell K.A., Liu S. (2015). MTOR regulates the pro-tumorigenic senescence-associated secretory phenotype by promoting IL1A translation. Nat. Cell Biol..

[37] Freund A., Patil C.K., Campisi J. (2011). p38MAPK is a novel DNA damage response-independent regulator of the senescence-associated secretory phenotype. EMBO J..

[38] Hou J., Cui C., Kim S., Sung C., Choi C. (2018). Ginsenoside F1 suppresses astrocytic senescence-associated secretory phenotype. Chem. Biol. Interact..

[39] Zhao J., Zhang L., Lu A., Han Y., Colangelo D., Bukata C., Scibetta A., Yousefzadeh M.J., Li X., Gurkar A.U. (2020). ATM is a key driver of NF-kB-dependent DNA-damage-induced senescence, stem cell dysfunction and aging. Aging.

[40] Chen C., Zhou M., Ge Y., Wang X. (2020). SIRT1 and aging related signaling pathways. Mech. Ageing Dev..

[41] Moiseeva O., Deschenes-Simard X., St-Germain E., Igelmann S., Huot G., Cadar A.E., Bourdeau V., Pollak M.N., Ferbeyre G. (2013). Metformin inhibits the senescence-associated secretory phenotype by interfering with IKK/NF-kappaB activation. Aging Cell.

[42] Perrott K.M., Wiley C.D., Desprez P.Y., Campisi J. (2017). Apigenin suppresses the senescence-associated secretory phenotype and paracrine effects on breast cancer cells. Geroscience.

[43] Pitozzi V., Mocali A., Laurenzana A., Giannoni E., Cifola I., Battaglia C., Chiarugi P., Dolara P., Giovannelli L. (2013). Chronic resveratrol treatment ameliorates cell adhesion and mitigates the inflammatory phenotype in senescent human fibroblasts. J. Gerontol. A Biol. Sci. Med. Sci..

[44] Xu M., Tchkonia T., Ding H., Ogrodnik M., Lubbers E.R., Pirtskhalava T., White T.A., Johnson K.O., Stout M.B., Mezera V. (2015). JAK inhibition alleviates the cellular senescence-associated secretory phenotype and frailty in old age. Proc. Natl. Acad. Sci. USA.

[45] Liu S., Uppal H., Demaria M., Desprez P.Y., Campisi J., Kapahi P. (2015). Simvastatin suppresses breast cancer cell proliferation induced by senescent cells. Sci. Rep..

[46] Crimmins E.M. (2015). Lifespan and healthspan: Past, present, and promise. Gerontologist.

[47] McPhail S.M. (2016). Multimorbidity in chronic disease: Impact on health care resources and costs. Risk Manag. Healthc. Policy.

[48] Noncommunicable Diseases. https://www.who.int/news-room/fact-sheets/detail/noncommunicable-diseases.

[49] Boccardi V., Mecocci P. (2021). Senotherapeutics: Targeting senescent cells for the main age-related diseases. Mech. Ageing Dev..

[50] Dimri G.P., Lee X.H., Basile G., Acosta M., Scott C., Roskelley C., Medrano E.E., Linskens M., Rubelj I., Pereirasmith O. (1995). A biomarker that identifies senescent human-cells in culture and in aging skin in-vivo. Proc. Natl. Acad. Sci. USA.

[51] Childs B.G., Baker D.J., Wijshake T., Conover C.A., Campisi J., van Deursen J.M. (2016). Senescent intimal foam cells are deleterious at all stages of atherosclerosis. Science.

[52] Muñoz-Espin D., Demaria M. (2020). Senolytics in Disease, Ageing and Longevity.

[53] Mongelli A., Atlante S., Barbi V., Bachetti T., Martelli F., Farsetti A., Gaetano C. (2020). Treating Senescence like Cancer: Novel Perspectives in Senotherapy of Chronic Diseases. Int. J. Mol. Sci..

[54] Borghesan M., Hoogaars W.M.H., Varela-Eirin M., Talma N., Demaria M. (2020). A senescence-centric view of aging: Implications for longevity and disease. Trends Cell Biol..

[55] He S., Sharpless N.E. (2017). Senescence in health and disease. Cell.

[56] Kirkland J.L., Tchkonia T. (2020). Senolytic drugs: From discovery to translation. J. Intern. Med..

[57] Roda A.R., Serra-Mir G., Montoliu-Gaya L., Tiessler L., Villegas S. (2022). Amyloid-beta peptide and tau protein crosstalk in Alzheimer’s disease. Neural Regen. Res..

[58] Armstrong M.J., Okun M.S. (2020). Diagnosis and treatment of parkinson disease a review. J. Am. Med. Assoc..

[59] Si Z.Z., Sun L.L., Wang X.D. (2021). Evidence and perspectives of cell senescence in neurodegenerative diseases. Biomed. Pharmacother..

[60] Bhat R., Crowe E.P., Bitto A., Moh M., Katsetos C.D., Garcia F.U., Johnson F.B., Trojanowski J.Q., Sell C., Torres C. (2012). Astrocyte senescence as a component of Alzheimer’s disease. PLoS ONE.

[61] Chinta S.J., Woods G., Demaria M., Rane A., Zou Y., McQuade A., Rajagopalan S., Limbad C., Madden D.T., Campisi J. (2018). Cellular senescence is induced by the environmental neurotoxin paraquat and contributes to neuropathology linked to Parkinson’s disease. Cell Rep..

[62] PerezGrovas-Saltijeral A., Ochoa-Morales A., Miranda-Duarte A., Martinez-Ruano L., Jara-Prado A., Camacho-Molina A., Hidalgo-Bravo A. (2020). Telomere length analysis on leukocytes derived from patients with Huntington Disease. Mech. Ageing Dev..

[63] Nicaise A.M., Wagstaff L.J., Willis C.M., Paisie C., Chandok H., Robson P., Fossati V., Williams A., Crocker S.J. (2019). Cellular senescence in progenitor cells contributes to diminished remyelination potential in progressive multiple sclerosis. Proc. Natl. Acad. Sci. USA.

[64] Birger A., Ben-Dor I., Ottolenghi M., Turetsky T., Gil Y., Sweetat S., Perez L., Belzer V., Casden N., Steiner D. (2019). Human iPSC-derived astrocytes from ALS patients with mutated C9ORF72 show increased oxidative stress and neurotoxicity. EBioMedicine.

[65] Jurk D., Wang C.F., Miwa S., Maddick M., Korolchuk V., Tsolou A., Gonos E.S., Thrasivoulou C., Saffrey M.J., Cameron K. (2012). Postmitotic neurons develop a p21-dependent senescence-like phenotype driven by a DNA damage response. Aging Cell.

[66] Musi N., Valentine J.M., Sickora K.R., Baeuerle E., Thompson C.S., Shen Q., Orr M.E. (2018). Tau protein aggregation is associated with cellular senescence in the brain. Aging Cell.

[67] Zhang P.S., Kishimoto Y., Grammatikakis I., Gottimukkala K., Cutler R.G., Zhang S.L., Abdelmohsen K., Bohr V.A., Sen J.M., Gorospe M. (2019). Senolytic therapy alleviates Abeta-associated oligodendrocyte progenitor cell senescence and cognitive deficits in an Alzheimer’s disease model. Nat. Neurosci..

[68] Han X., Zhang T., Liu H., Mi Y., Gou X. (2020). Astrocyte senescence and alzheimer’s disease: A review. Front. Aging Neurosci..

[69] Ogrodnik M., Evans S.A., Fielder E., Victorelli S., Kruger P., Salmonowicz H., Weigand B.M., Patel A.D., Pirtskhalava T., Inman C.L. (2021). Whole-body senescent cell clearance alleviates age-related brain inflammation and cognitive impairment in mice. Aging Cell.

[70] Elsallabi O., Patruno A., Pesce M., Cataldi A., Carradori S., Gallorini M. (2022). Fisetin as a senotherapeutic agent: Biopharmaceutical properties and crosstalk between cell senescence and neuroprotection. Molecules.

[71] Bussian T.J., Aziz A., Meyer C.F., Swenson B.L., van Deursen J.M., Baker D.J. (2018). Clearance of senescent glial cells prevents tau-dependent pathology and cognitive decline. Nature.

[72] Go J., Ha T.K.Q., Seo J.Y., Park T.S., Ryu Y.K., Park H.Y., Noh J.R., Kim Y.H., Hwang J.H., Choi D.H. (2018). Piperlongumine activates Sirtuin1 and improves cognitive function in a murine model of Alzheimer’s disease. J. Funct. Foods.

[73] Katila N., Bhurtel S., Shadfar S., Srivastav S., Neupane S., Ojha U., Jeong G.S., Choi D.Y. (2017). Metformin lowers alpha-synuclein phosphorylation and upregulates neurotrophic factor in the MPTP mouse model of Parkinson’s disease. Neuropharmacology.

[74] Van Skike C.E., Jahrling J.B., Olson A.B., Sayre N.L., Hussong S.A., Ungvari Z., Lechleiter J.D., Galvan V. (2018). Inhibition of mTOR protects the blood-brain barrier in models of Alzheimer’s disease and vascular cognitive impairment. Am. J. Physiol.-Heart C.

[75] Tang Z., Bereczki E., Zhang H.Y., Wang S., Li C.X., Ji X.Y., Branca R.M., Lehtio J., Guan Z.Z., Filipcik P. (2013). Mammalian target of rapamycin (mtor) mediates tau protein dyshomeostasis implicaton fo alzheimer’s disease. J. Biol. Chem..

[76] Ou Z.R., Kong X.J., Sun X.D., He X.S., Zhang L., Gong Z., Huang J.Y., Xu B.A., Long D.H., Li J.H. (2018). Metformin treatment prevents amyloid plaque deposition and memory impairment in APP/PS1 mice. Brain Behav. Immun..

[77] Gonzales M.M., Krishnamurthy S., Garbarino V., Daeihagh A.S., Gillispie G.J., Deep G., Craft S., Orr M.E. (2021). A geroscience motivated approach to treat Alzheimer’s disease: Senolytics move to clinical trials. Mech. Ageing Dev..

[78] Hamsanathan S., Alder J.K., Sellares J., Rojas M., Gurkar A.U., Mora A.L. (2019). Cellular senescence: The trojan horse in chronic lung diseases. Am. J. Respir. Cell Mol. Biol..

[79] Liu R.M., Liu G. (2020). Cell senescence and fibrotic lung diseases. Exp. Gerontol..

[80] Araya J., Kuwano K. (2022). Cellular senescence-an aging hallmark in chronic obstructive pulmonary disease pathogenesis. Respir. Investig..

[81] Barnes P.J. (2021). Targeting cellular senescence as a new approach to chronic obstructive pulmonary disease therapy. Curr. Opin. Pharmacol..

[82] Tuder R.M. (2006). Aging and cigarette smoke: Fueling the fire. Am. J. Respir. Crit. Care Med..

[83] Leung J., Cho Y., Lockey R.F., Kolliputi N. (2015). The role of aging in idiopathic pulmonary fibrosis. Lung.

[84] Alvarez D., Cardenes N., Sellares J., Bueno M., Corey C., Hanumanthu V.S., Peng Y.T., D’Cunha H., Sembrat J., Nouraie M. (2017). IPF lung fibroblasts have a senescent phenotype. Am. J. Physiol.-Lung C.

[85] Schafer M.J., White T.A., Iijima K., Haak A.J., Ligresti G., Atkinson E.J., Oberg A.L., Birch J., Salmonowicz H., Zhu Y. (2017). Cellular senescence mediates fibrotic pulmonary disease. Nat. Commun..

[86] Muller K.C., Welker L., Paasch K., Feindt B., Erpenbeck V., Hohlfeld J., Krug N., Nakashima M., Branscheid D., Magnussen H. (2006). Lung fibroblasts from patients with emphysema show markers of senescence in vitro. Resp. Res..

[87] Lehmann M., Korfei M., Mutze K., Klee S., Skronska-Wasek W., Alsafadi H.N., Ota C., Costa R., Schiller H.B., Lindner M. (2017). Senolytic drugs target alveolar epithelial cell function and attenuate experimental lung fibrosis ex vivo. Eur. Resp. J..

[88] Hohmann M.S., Habiel D.M., Coelho A.L., Verri W.A., Hogaboam C.M. (2019). Quercetin enhances ligand-induced apoptosis in senescent idiopathic pulmonary fibrosis fibroblasts and reduces lung fibrosis in vivo. Am. J. Resp. Cell Mol. Biol..

[89] Tsuji T., Aoshiba K., Nagai A. (2006). Alveolar cell senescence in patients with pulmonary emphysema. Am. J. Resp. Crit. Care Med..

[90] Baker J.R., Donnelly L.E., Barnes P.J. (2020). Senotherapy a new horizon for COPD therapy. Chest.

[91] Lamming D.W., Ye L., Sabatini D.M., Baur J.A. (2013). Rapalogs and mTOR inhibitors as anti-aging therapeutics. J. Clin. Investig..

[92] Polverino F., Wu T.D., Rojas-Quintero J., Wang X.Y., Mayo J., Tomchaney M., Tram J., Packard S., Zhang D., Cleveland K.H. (2021). Metformin: Experimental and clinical evidence for a potential role in emphysema treatment. Am. J. Resp. Crit. Care Med..

[93] Hubbard B.P., Sinclair D.A. (2014). Small molecule SIRT1 activators for the treatment of aging and age-related diseases. Trends Pharmacol. Sci..

[94] Easter M., Bollenbecker S., Barnes J.W., Krick S. (2020). Targeting aging pathways in chronic obstructive pulmonary disease. Int. J. Mol. Sci..

[95] Yen F.S., Chen W.S., Wei J.C.C., Hsu C.C., Hwu C.M. (2018). Effects of metformin use on total mortality in patients with type 2 diabetes and chronic obstructive pulmonary disease: A matched-subject design. PLoS ONE.

[96] Roth G.A., Mensah G.A., Johnson C.O., Addolorato G., Ammirati E., Baddour L.M., Barengo N.C., Beaton A.Z., Benjamin E.J., Benziger C.P. (2020). Global burden of cardiovascular diseases and risk factors, 1990–2019 update from the GBD 2019 study. J. Am. Coll. Cardiol..

[97] Wang J.C., Bennett M. (2012). Aging and atherosclerosis mechanisms, functional consequences, and potential therapeutics for cellular senescence. Circ. Res..

[98] Lewis-McDougall F.C., Ruchaya P.J., Domenjo-Vila E., Teoh T.S., Prata L., Cottle B.J., Clark J.E., Punjabi P.P., Awad W., Torella D. (2019). Aged-senescent cells contribute to impaired heart regeneration. Aging Cell.

[99] Anderson R., Lagnado A., Maggiorani D., Walaszczyk A., Dookun E., Chapman J., Birch J., Salmonowicz H., Ogrodnik M., Jurk D. (2019). Length-independent telomere damage drives post-mitotic cardiomyocyte senescence. EMBO J..

[100] El Hadri K., Smith R., Duplus E., El Amri C. (2022). Inflammation, oxidative stress, senescence in atherosclerosis: Thioredoxine-1 as an emerging therapeutic target. Int. J. Mol. Sci..

[101] Hamczyk M.R., Villa-Bellosta R., Quesada V., Gonzalo P., Vidak S., Nevado R.M., Andrés-Manzano M.J., Misteli T., López-Otín C., Andrés V. (2019). Progerin accelerates atherosclerosis by inducing endoplasmic reticulum stress in vascular smooth muscle cells. EMBO Mol. Med..

[102] Macicior J., Marcos-Ramiro B., Ortega-Gutiérrez S. (2021). Small-molecule therapeutic perspectives for the treatment of progeria. Int. J. Mol. Sci..

[103] Marcos-Ramiro B., Gil-Ordóñez A., Marín-Ramos N.I., Ortega-Nogales F.J., Balabasquer M., Gonzalo P., Khiar-Fernández N., Rolas L., Barkaway A., Nourshargh S. (2021). Isoprenylcysteine carboxylmethyltransferase-based therapy for Hutchinson-Gilford progeria syndrome. ACS Cent. Sci..

[104] Walaszczyk A., Dookun E., Redgrave R., Tual-Chalot S., Victorelli S., Spyridopoulos I., Owens A., Arthur H.M., Passos J.F., Richardson G.D. (2019). Pharmacological clearance of senescent cells improves survival and recovery in aged mice following acute myocardial infarction. Aging Cell.

[105] Roos C.M., Zhang B., Palmer A.K., Ogrodnik M.B., Pirtskhalava T., Thalji N.M., Hagler M., Jurk D., Smith L.A., Casaclang-Verzosa G. (2016). Chronic senolytic treatment alleviates established vasomotor dysfunction in aged or atherosclerotic mice. Aging Cell.

[106] Owens W.A., Walaszczyk A., Spyridopoulos I., Dookun E., Richardson G.D. (2021). Senescence and senolytics in cardiovascular disease: Promise and potential pitfalls. Mech. Ageing Dev..

[107] Sweeney M., Cook S.A., Gil J. (2022). Therapeutic opportunities for senolysis in cardiovascular disease. FEBS J..

[108] Shimizu I., Minamino T. (2019). Cellular senescence in cardiac diseases. J. Cardiol..

[109] Diabetes. https://www.who.int/health-topics/diabetes-tab=tab_1.

[110] Palmer A.K., Gustafson B., Kirkland J.L., Smith U. (2019). Cellular senescence: At the nexus between ageing and diabetes. Diabetologia.

[111] Palmer A.K., Xu M., Zhu Y., Pirtskhalava T., Weivoda M.M., Hachfeld C.M., Prata L.G., van Dijk T.H., Verkade E., Casaclang-Verzosa G. (2019). Targeting senescent cells alleviates obesity-induced metabolic dysfunction. Aging Cell.

[112] Aguayo-Mazzucato C., Andle J., Lee T.B., Midha A., Talemal L., Chipashvili V., Hollister-Lock J., van Deursen J., Weir G., Bonner-Weir S. (2019). Acceleration of beta cell aging determines diabetes and senolysis improves disease outcomes. Cell Metab..

[113] Xu M., Pirtskhalava T., Farr J.N., Weigand B.M., Palmer A.K., Weivoda M.M., Inman C.L., Ogrodnik M.B., Hachfeld C.M., Fraser D.G. (2018). Senolytics improve physical function and increase lifespan in old age. Nat. Med..

[114] Yousefzadeh M.J., Zhu Y., McGowan S.J., Angelini L., Fuhrmann-Stroissnigg H., Xu M., Ling Y.Y., Melos K.I., Pirtskhalava T., Inman C.L. (2018). Fisetin is a senotherapeutic that extends health and lifespan. Ebiomedicine.

[115] Thompson P.J., Shah A., Ntranos V., Van Gool F., Atkinson M., Bhushan A. (2019). Targeted elimination of senescent beta cells prevents type 1 diabetes. Cell Metab..

[116] Khosla S., Farr J.N., Tchkonia T., Kirkland J.L. (2020). The role of cellular senescence in ageing and endocrine disease. Nat. Rev. Endocrinol..

[117] Barzilai N.R. (2017). Targeting aging with metformin (TAME). Innov. Aging.

[118] Kloppenburg M., Berenbaum F. (2020). Osteoarthritis year in review 2019: Epidemiology and therapy. Osteoarth. Cartil..

[119] Cruz-Jentoft A.J., Sayer A.A. (2019). Sarcopenia. Lancet.

[120] Cao X., Luo P., Huang J.J., Liang C., He J.S., Wang Z.L., Shan D.Y., Peng C., Wu S. (2019). Intraarticular senescent chondrocytes impair the cartilage regeneration capacity of mesenchymal stem cells. Stem Cell Res. Ther..

[121] Farr J.N., Fraser D.G., Wang H., Jaehn K., Ogrodnik M.B., Weivoda M.M., Drake M.T., Tchkonia T., LeBrasseur N.K., Kirkland J.L. (2016). Identification of senescent cells in the bone microenvironment. J. Bone Miner. Res..

[122] Saito Y., Chikenji T.S., Matsumura T., Nakano M., Fujimiya M. (2020). Exercise enhances skeletal muscle regeneration by promoting senescence in fibro-adipogenic progenitors. Nat. Commun..

[123] Farr J.N., Xu M., Weivoda M.M., Monroe D.G., Fraser D.G., Onken J.L., Negley B.A., Sfeir J.G., Ogrodnik M.B., Hachfeld C.M. (2017). Targeting cellular senescence prevents age-related bone loss in mice. Nat. Med..

[124] Li J., Zhang B., Liu W.X., Lu K., Pan H.B., Wang T.Y., Oh C.D., Yi D., Huang J., Zhao L. (2020). Metformin limits osteoarthritis development and progression through activation of AMPK signalling. Ann. Rheum. Dis..

[125] Takayama K., Kawakami Y., Kobayashi M., Greco N., Cummins J.H., Matsushita T., Kuroda R., Kurosaka M., Fu F.H., Huard J. (2014). Local intra-articular injection of rapamycin delays articular cartilage degeneration in a murine model of osteoarthritis. Arthritis Res. Ther..

[126] Wan M., Gray-Gaillard E.F., Elisseeff J.H. (2021). Cellular senescence in musculoskeletal homeostasis, diseases, and regeneration. Bone Res..

[127] Ferreira-Gonzalez S., Rodrigo-Torres D., Gadd V.L., Forbes S.J. (2021). Cellular senescence in liver disease and regeneration. Sem. Liver Dis..

[128] Meijnikman A.S., Herrema H., Scheithauer T.P.M., Kroon J., Nieuwdorp M., Groen A.K. (2021). Evaluating causality of cellular senescence in non-alcoholic fatty liver disease. JHEP Rep..

[129] Wang W.J., Cai G.Y., Chen X.M. (2017). Cellular senescence, senescence-associated secretory phenotype, and chronic kidney disease. Oncotarget.

[130] Zhou B.R., Wan Y., Chen R., Zhang C.M., Li X.S., Meng F.Y., Glaser S., Wu N., Zhou T.H., Li S.W. (2020). The emerging role of cellular senescence in renal diseases. J. Cell. Mol. Med..

[131] Sreekumar P.G., Hinton D.R., Kannan R. (2020). The emerging role of senescence in ocular disease. Oxid. Med. Cell. Longev..

[132] Sargiacomo C., Sotgia F., Lisanti M.P. (2020). COVID-19 and chronological aging: Senolytics and other anti-aging drugs for the treatment or prevention of corona virus infection?. Aging-US.

[133] Verdoorn B.P., Evans T.K., Hanson G.J., Zhu Y., Prata L.G.P.L., Pignolo R.J., Atkinson E.J., Wissler-Gerdes E.O., Kuchel G.A., Mannick J.B. (2021). Fisetin for COVID-19 in skilled nursing facilities: Senolytic trials in the COVID era. J. Am. Geriatr. Soc..

[134] Partridge L., Fuentealba M., Kennedy B.K. (2020). The quest to slow ageing through drug discovery. Nat. Rev. Drug Discov..

[135] Formica J.V., Regelson W. (1995). Review of the biology of quercetin and related bioflavonoids. Food Chem. Toxicol..

[136] Zhu Y., Tchkonia T., Pirtskhalava T., Gower A.C., Ding H., Giorgadze N., Palmer A.K., Ikeno Y., Hubbard G.B., Lenburg M. (2015). The Achilles’ heel of senescent cells: From transcriptome to senolytic drugs. Aging Cell.

[137] Ogrodnik M., Miwa S., Tchkonia T., Tiniakos D., Wilson C.L., Lahat A., Day C.P., Burt A., Palmer A., Anstee Q.M. (2017). Cellular senescence drives age-dependent hepatic steatosis. Nat. Commun..

[138] Ogrodnik M., Zhu Y., Langhi L.G.P., Tchkonia T., Krüger P., Fielder E., Victorelli S., Ruswhandi R.A., Giorgadze N., Pirtskhalava T. (2019). Obesity-induced cellular senescence drives anxiety and impairs neurogenesis. Cell Metab..

[139] Sundarraj K., Raghunath A., Perumal E. (2018). A review on the chemotherapeutic potential of fisetin: In vitro evidences. Biomed. Pharmacother..

[140] Syed D.N., Adhami V.M., Khan N., Khan M.I., Mukhtar H. (2016). Exploring the molecular targets of dietary flavonoid fisetin in cancer. Semin. Cancer Biol..

[141] Yang D., Tian X., Ye Y., Liang Y., Zhao J., Wu T., Lu N. (2021). Identification of GL-V9 as a novel senolytic agent against senescent breast cancer cells. Life Sci..

[142] Li H., Hu P., Wang Z., Wang H., Yu X., Wang X., Qing Y., Zhu M., Xu J., Li Z. (2020). Mitotic catastrophe and p53-dependent senescence induction in T-cell malignancies exposed to nonlethal dosage of GL-V9. Arch. Toxicol..

[143] Wang Y., Chang J., Liu X., Zhang X., Zhang S., Zhang X., Zhou D., Zheng G. (2016). Discovery of piperlongumine as a potential novel lead for the development of senolytic agents. Aging.

[144] Zhang X., Zhang S., Liu X., Wang Y., Chang J., Zhang X., Mackintosh S.G., Tackett A.J., He Y., Lv D. (2018). Oxidation resistance 1 is a novel senolytic target. Aging Cell.

[145] Liu X., Wang Y., Zhang X., Gao Z., Zhang S., Shi P., Zhang X., Song L., Hendrickson H., Zhou D. (2018). Senolytic activity of piperlongumine analogues: Synthesis and biological evaluation. Bioorg. Med. Chem..

[146] Hewlings S.J., Kalman D.S. (2017). Curcumin: A review of its effects on human health. Foods.

[147] Cherif H., Bisson D.G., Jarzem P., Weber M., Ouellet J.A., Haglund L. (2019). Curcumin and o-vanillin exhibit evidence of senolytic activity in human IVD Cells in vitro. J. Clin. Med..

[148] Li W., He Y., Zhang R., Zheng G., Zhou D. (2019). The curcumin analog EF24 is a novel senolytic agent. Aging.

[149] Triana-Martínez F., Picallos-Rabina P., Da Silva-Álvarez S., Pietrocola F., Llanos S., Rodilla V., Soprano E., Pedrosa P., Ferreirós A., Barradas M. (2019). Identification and characterization of cardiac glycosides as senolytic compounds. Nat. Commun..

[150] Guerrero A., Herranz N., Sun B., Wagner V., Gallage S., Guiho R., Wolter K., Pombo J., Irvine E.E., Innes A.J. (2019). Cardiac glycosides are broad-spectrum senolytics. Nat. Metab..

[151] Tse C., Shoemaker A.R., Adickes J., Anderson M.G., Chen J., Jin S., Johnson E.F., Marsh K.C., Mitten M.J., Nimmer P. (2008). ABT-263: A potent and orally bioavailable Bcl-2 family inhibitor. Cancer Res..

[152] Pan J., Li D., Xu Y., Zhang J., Wang Y., Chen M., Lin S., Huang L., Chung E.J., Citrin D.E. (2017). Inhibition of Bcl-2/xl With ABT-263 Selectively Kills Senescent Type II Pneumocytes and Reverses Persistent Pulmonary Fibrosis Induced by Ionizing Radiation in Mice. Int. J. Radiat. Oncol. Biol. Phys..

[153] Mellatyar H., Talaei S., Pilehvar-Soltanahmadi Y., Barzegar A., Akbarzadeh A., Shahabi A., Barekati-Mowahed M., Zarghami N. (2018). Targeted cancer therapy through 17-DMAG as an Hsp90 inhibitor: Overview and current state of the art. Biomed. Pharmacother..

[154] Morsli S., Doherty G.J., Muñoz-Espín D. (2022). Activatable senoprobes and senolytics: Novel strategies to detect and target senescent cells. Mech. Ageing Dev..

[155] González-Gualda E., Pàez-Ribes M., Lozano-Torres B., Macias D., Wilson J.R., González-López C., Ou H.-L., Mirón-Barroso S., Zhang Z., Lérida-Viso A. (2020). Galacto-conjugation of Navitoclax as an efficient strategy to increase senolytic specificity and reduce platelet toxicity. Aging Cell.

[156] Galiana I., Lozano-Torres B., Sancho M., Alfonso M., Bernardos A., Bisbal V., Serrano M., Martínez-Máñez R., Orzáez M. (2020). Preclinical antitumor efficacy of senescence-inducing chemotherapy combined with a nanoSenolytic. J. Control. Release.

[157] He Y., Zhang X., Chang J., Kim H.-N., Zhang P., Wang Y., Khan S., Liu X., Zhang X., Lv D. (2020). Using proteolysis-targeting chimera technology to reduce navitoclax platelet toxicity and improve its senolytic activity. Nat. Commun..

[158] Vézina C., Kudelski A., Sehgal S.N. (1975). Rapamycin (AY-22,989), a new antifungal antibiotic. I. Taxonomy of the producing streptomycete and isolation of the active principle. J. Antibiot..

[159] Flynn J.M., O’Leary M.N., Zambataro C.A., Academia E.C., Presley M.P., Garrett B.J., Zykovich A., Mooney S.D., Strong R., Rosen C.J. (2013). Late-life rapamycin treatment reverses age-related heart dysfunction. Aging Cell.

[160] Majumder S., Caccamo A., Medina D.X., Benavides A.D., Javors M.A., Kraig E., Strong R., Richardson A., Oddo S. (2012). Lifelong rapamycin administration ameliorates age-dependent cognitive deficits by reducing IL-1β and enhancing NMDA signaling. Aging Cell.

[161] Bjedov I., Rallis C. (2020). The target of rapamycin signalling pathway in ageing and lifespan regulation. Genes.

[162] Wang R., Yu Z., Sunchu B., Shoaf J., Dang I., Zhao S., Caples K., Bradley L., Beaver L.M., Ho E. (2017). Rapamycin inhibits the secretory phenotype of senescent cells by a Nrf2-independent mechanism. Aging Cell.

[163] Li J., Kim S.G., Blenis J. (2014). Rapamycin: One Drug, many effects. Cell Metab..

[164] Lim H., Park H., Kim H.P. (2015). Effects of flavonoids on senescence-associated secretory phenotype formation from bleomycin-induced senescence in BJ fibroblasts. Biochem. Pharmacol..

[165] Harrison D.E., Strong R., Allison D.B., Ames B.N., Astle C.M., Atamna H., Fernandez E., Flurkey K., Javors M.A., Nadon N.L. (2014). Acarbose, 17-α-estradiol, and nordihydroguaiaretic acid extend mouse lifespan preferentially in males. Aging Cell.

[166] Campbell J.M., Bellman S.M., Stephenson M.D., Lisy K. (2017). Metformin reduces all-cause mortality and diseases of ageing independent of its effect on diabetes control: A systematic review and meta-analysis. Ageing Res. Rev..

[167] Bannister C.A., Holden S.E., Jenkins-Jones S., Morgan C.L., Halcox J.P., Schernthaner G., Mukherjee J., Currie C.J. (2014). Can people with type 2 diabetes live longer than those without? A comparison of mortality in people initiated with metformin or sulphonylurea monotherapy and matched, non-diabetic controls. Diabetes Obes. Metab..

[168] The TAME Trial. https://www.afar.org/tame-trial.

[169] Tilstra J.S., Robinson A.R., Wang J., Gregg S.Q., Clauson C.L., Reay D.P., Nasto L.A., St Croix C.M., Usas A., Vo N. (2012). NF-κB inhibition delays DNA damage–induced senescence and aging in mice. J. Clin. Investig..

[170] Zhang L., Zhao J., Mu X., McGowan S.J., Angelini L., O’Kelly R.D., Yousefzadeh M.J., Sakamoto A., Aversa Z., LeBrasseur N.K. (2021). Novel small molecule inhibition of IKK/NF-κB activation reduces markers of senescence and improves healthspan in mouse models of aging. Aging Cell.

[171] Alimbetov D., Davis T., Brook A.J.C., Cox L.S., Faragher R.G.A., Nurgozhin T., Zhumadilov Z., Kipling D. (2016). Suppression of the senescence-associated secretory phenotype (SASP) in human fibroblasts using small molecule inhibitors of p38 MAP kinase and MK2. Biogerontology.

[172] Griveau A., Wiel C., Ziegler D.V., Bergo M.O., Bernard D. (2020). The JAK1/2 inhibitor ruxolitinib delays premature aging phenotypes. Aging Cell.

[173] Kang H.T., Park J.T., Choi K., Kim Y., Choi H.J.C., Jung C.W., Lee Y.-S., Park S.C. (2017). Chemical screening identifies ATM as a target for alleviating senescence. Nat. Chem. Biol..

[174] Kuk M.U., Kim J.W., Lee Y., Cho K.A., Park J.T., Park S.C. (2019). Alleviation of senescence via ATM inhibition in accelerated aging models. Mol. Cells.

[175] Liu J., Jiao K., Zhou Q., Yang J., Yang K., Hu C., Zhou M., Li Z. (2021). Resveratrol alleviates 27-hydroxycholesterol-induced senescence in nerve cells and affects zebrafish locomotor behavior via activation of SIRT1-mediated STAT3 signaling. Oxid. Med. Cell. Longev..

[176] Csiszar A., Sosnowska D., Wang M., Lakatta E.G., Sonntag W.E., Ungvari Z. (2012). Age-associated proinflammatory secretory phenotype in vascular smooth muscle cells from the non-human primate *Macaca mulatta*: Reversal by resveratrol treatment. J. Gerontol. A Biol. Sci. Med. Sci..

[177] Mitchell S.J., Martin-Montalvo A., Mercken E.M., Palacios H.H., Ward T.M., Abulwerdi G., Minor R.K., Vlasuk G.P., Ellis J.L., Sinclair D.A. (2014). The SIRT1 activator SRT1720 extends lifespan and improves health of mice fed a standard diet. Cell Rep..

[178] Conboy M.J., Conboy I.M., Rando T.A. (2013). Heterochronic parabiosis: Historical perspective and methodological considerations for studies of aging and longevity. Aging Cell.

[179] Yousefzadeh M.J., Wilkinson J.E., Hughes B., Gadela N., Ladiges W.C., Vo N., Niedernhofer L.J., Huffman D.M., Robbins P.D. (2020). Heterochronic parabiosis regulates the extent of cellular senescence in multiple tissues. Geroscience.

[180] Conboy I.M., Conboy M.J., Wagers A.J., Girma E.R., Weissman I.L., Rando T.A. (2005). Rejuvenation of aged progenitor cells by exposure to a young systemic environment. Nature.

[181] Ruckh J.M., Zhao J.W., Shadrach J.L., van Wijngaarden P., Rao T.N., Wagers A.J., Franklin R.J. (2012). Rejuvenation of regeneration in the aging central nervous system. Cell Stem Cell.

[182] Villeda S.A., Luo J., Mosher K.I., Zou B., Britschgi M., Bieri G., Stan T.M., Fainberg N., Ding Z., Eggel A. (2011). The ageing systemic milieu negatively regulates neurogenesis and cognitive function. Nature.

[183] Villeda S.A., Plambeck K.E., Middeldorp J., Castellano J.M., Mosher K.I., Luo J., Smith L.K., Bieri G., Lin K., Berdnik D. (2014). Young blood reverses age-related impairments in cognitive function and synaptic plasticity in mice. Nat. Med..

[184] Huang Q., Ning Y., Liu D., Zhang Y., Li D., Zhang Y., Yin Z., Fu B., Cai G., Sun X. (2018). A young blood environment decreases aging of senile mice kidneys. J. Gerontol. A Biol. Sci. Med. Sci..

[185] Salpeter S.J., Khalaileh A., Weinberg-Corem N., Ziv O., Glaser B., Dor Y. (2013). Systemic regulation of the age-related decline of pancreatic β-cell replication. Diabetes.

[186] Baht G.S., Silkstone D., Vi L., Nadesan P., Amani Y., Whetstone H., Wei Q., Alman B.A. (2015). Exposure to a youthful circulation rejuvenates bone repair through modulation of β-catenin. Nat. Commun..

[187] Kiss T., Nyúl-Tóth Á., Gulej R., Tarantini S., Csipo T., Mukli P., Ungvari A., Balasubramanian P., Yabluchanskiy A., Benyo Z. (2022). Old blood from heterochronic parabionts accelerates vascular aging in young mice: Transcriptomic signature of pathologic smooth muscle remodeling. Geroscience.

[188] Kiss T., Tarantini S., Csipo T., Balasubramanian P., Nyúl-Tóth Á., Yabluchanskiy A., Wren J.D., Garman L., Huffman D.M., Csiszar A. (2020). Circulating anti-geronic factors from heterochonic parabionts promote vascular rejuvenation in aged mice: Transcriptional footprint of mitochondrial protection, attenuation of oxidative stress, and rescue of endothelial function by young blood. Geroscience.

[189] Smith L.K., He Y., Park J.S., Bieri G., Snethlage C.E., Lin K., Gontier G., Wabl R., Plambeck K.E., Udeochu J. (2015). β2-microglobulin is a systemic pro-aging factor that impairs cognitive function and neurogenesis. Nat. Med..

[190] Li R., Liang Y., Lin B. (2022). Accumulation of systematic TPM1 mediates inflammation and neuronal remodeling by phosphorylating PKA and regulating the FABP5/NF-κB signaling pathway in the retina of aged mice. Aging Cell.

[191] Castellano J.M., Mosher K.I., Abbey R.J., McBride A.A., James M.L., Berdnik D., Shen J.C., Zou B., Xie X.S., Tingle M. (2017). Human umbilical cord plasma proteins revitalize hippocampal function in aged mice. Nature.

[192] Iram T., Kern F., Kaur A., Myneni S., Morningstar A.R., Shin H., Garcia M.A., Yerra L., Palovics R., Yang A.C. (2022). Young CSF restores oligodendrogenesis and memory in aged mice via Fgf17. Nature.

[193] Smith L.K., Verovskaya E., Bieri G., Horowitz A.M., von Ungern-Sternberg S.N.I., Lin K., Seizer P., Passegué E., Villeda S.A. (2020). The aged hematopoietic system promotes hippocampal-dependent cognitive decline. Aging Cell.

[194] Ho T.T., Dellorusso P.V., Verovskaya E.V., Bakker S.T., Flach J., Smith L.K., Ventura P.B., Lansinger O.M., Hérault A., Zhang S.Y. (2021). Aged hematopoietic stem cells are refractory to bloodborne systemic rejuvenation interventions. J. Exp. Med..

[195] Levy M., Kolodziejczyk A.A., Thaiss C.A., Elinav E. (2017). Dysbiosis and the immune system. Nat. Rev. Immunol..

[196] Chaudhari S.N., McCurry M.D., Devlin A.S. (2021). Chains of evidence from correlations to causal molecules in microbiome-linked diseases. Nat. Chem. Biol..

[197] Helmink B.A., Khan M.A.W., Hermann A., Gopalakrishnan V., Wargo J.A. (2019). The microbiome, cancer, and cancer therapy. Nat. Med..

[198] Papadopoulos P.D., Tsigalou C., Valsamaki P.N., Konstantinidis T.G., Voidarou C., Bezirtzoglou E. (2022). The emerging role of the gut microbiome in cardiovascular disease: Current knowledge and perspectives. Biomedicines.

[199] Vallianou N., Christodoulatos G.S., Karampela I., Tsilingiris D., Magkos F., Stratigou T., Kounatidis D., Dalamaga M. (2022). Understanding the role of the gut microbiome and microbial metabolites in non-alcoholic fatty liver disease: Current evidence and perspectives. Biomolecules.

[200] Zhou Y., Hu G., Wang M.C. (2021). Host and microbiota metabolic signals in aging and longevity. Nat. Chem. Biol..

[201] Claesson M.J., Jeffery I.B., Conde S., Power S.E., O’Connor E.M., Cusack S., Harris H.M., Coakley M., Lakshminarayanan B., O’Sullivan O. (2012). Gut microbiota composition correlates with diet and health in the elderly. Nature.

[202] Shin J., Noh J.R., Choe D., Lee N., Song Y., Cho S., Kang E.J., Go M.J., Ha S.K., Chang D.H. (2021). Ageing and rejuvenation models reveal changes in key microbial communities associated with healthy ageing. Microbiome.

[203] Bárcena C., Valdés-Mas R., Mayoral P., Garabaya C., Durand S., Rodríguez F., Fernández-García M.T., Salazar N., Nogacka A.M., Garatachea N. (2019). Healthspan and lifespan extension by fecal microbiota transplantation into progeroid mice. Nat. Med..

[204] Biagi E., Franceschi C., Rampelli S., Severgnini M., Ostan R., Turroni S., Consolandi C., Quercia S., Scurti M., Monti D. (2016). Gut microbiota and extreme longevity. Curr. Biol..

[205] Sato Y., Atarashi K., Plichta D.R., Arai Y., Sasajima S., Kearney S.M., Suda W., Takeshita K., Sasaki T., Okamoto S. (2021). Novel bile acid biosynthetic pathways are enriched in the microbiome of centenarians. Nature.

[206] Borgoni S., Kudryashova K.S., Burka K., de Magalhães J.P. (2021). Targeting immune dysfunction in aging. Ageing Res. Rev..

[207] Cunha L.L., Perazzio S.F., Azzi J., Cravedi P., Riella L.V. (2020). Remodeling of the immune response with aging: Immunosenescence and its potential impact on COVID-19 immune response. Front. Immunol..

[208] Yoshida S., Nakagami H., Hayashi H., Ikeda Y., Sun J., Tenma A., Tomioka H., Kawano T., Shimamura M., Morishita R. (2020). The CD153 vaccine is a senotherapeutic option for preventing the accumulation of senescent T cells in mice. Nat. Commun..

[209] Martínez P., Blasco M.A. (2017). Telomere-driven diseases and telomere-targeting therapies. J. Cell Biol..

[210] Fischer F., Grigolon G., Benner C., Ristow M. (2022). Evolutionarily conserved transcription factors as regulators of longevity and targets for geroprotection. Physiol. Rev..

[211] Secci R., Hartmann A., Walter M., Grabe H.J., Van der Auwera-Palitschka S., Kowald A., Palmer D., Rimbach G., Fuellen G., Barrantes I. (2021). Biomarkers of geroprotection and cardiovascular health: An overview of omics studies and established clinical biomarkers in the context of diet. Crit. Rev. Food Sci. Nutr..

[212] Van Praag H., Shubert T., Zhao C., Gage F.H. (2005). Exercise enhances learning and hippocampal neurogenesis in aged mice. J. Neurosci..

[213] Speisman R.B., Kumar A., Rani A., Foster T.C., Ormerod B.K. (2013). Daily exercise improves memory, stimulates hippocampal neurogenesis and modulates immune and neuroimmune cytokines in aging rats. Brain Behav. Immun..

[214] Horowitz A.M., Fan X., Bieri G., Smith L.K., Sanchez-Diaz C.I., Schroer A.B., Gontier G., Casaletto K.B., Kramer J.H., Williams K.E. (2020). Blood factors transfer beneficial effects of exercise on neurogenesis and cognition to the aged brain. Science.

